# Astrocyte control of the entorhinal cortex‐dentate gyrus circuit: Relevance to cognitive processing and impairment in pathology

**DOI:** 10.1002/glia.24128

**Published:** 2021-12-14

**Authors:** Maria Amalia Di Castro, Andrea Volterra

**Affiliations:** ^1^ Department of Fundamental Neuroscience University of Lausanne Lausanne Switzerland; ^2^ Department of Physiology and Pharmacology Sapienza University of Rome Rome Italy

**Keywords:** astrocyte, cognitive impairment, gliotransmission, hippocampal memory, presynaptic NMDA receptors, synaptic modulation, TNF alpha

## Abstract

The entorhinal cortex‐dentate gyrus circuit is centrally involved in memory processing conveying to the hippocampus spatial and nonspatial context information via, respectively, medial and lateral perforant path (MPP and LPP) excitatory projections onto dentate granule cells (GCs). Here, we review work of several years from our group showing that astrocytes sense local synaptic transmission and exert in turn a presynaptic control at PP‐GC synapses. Modulation of neurotransmitter release probability by astrocytes sets basal synaptic strength and dynamic range for long‐term potentiation of PP‐GC synapses. Intriguingly, this astrocyte control is circuit‐specific, being present only at MPP‐GC (not LPP‐GC) synapses, which selectively express atypical presynaptic N‐methyl‐D‐aspartate receptors (NMDAR) suitable to activation by astrocyte‐released glutamate. Moreover, the astrocytic control is peculiarly dependent on the cytokine TNFα, which at constitutive levels acts as a gating factor for the astrocyte signaling. During inflammation/infection processes, increased levels of TNFα lead to uncontrolled astrocyte glutamate release, altered PP‐GC circuit processing and, ultimately, impaired contextual memory performance. The TNFα‐dependent pathological switch of the synaptic control from astrocytes and its deleterious consequences are observed in animal models of HIV brain infection and multiple sclerosis, conditions both known to cause cognitive disturbances in up to 50% of patients. The review also discusses open issues related to the identified astrocytic pathway: its role in contextual memory processing, potential damaging role in Alzheimer's disease, the existence of vesicular glutamate release from DG astrocytes, and the possible synaptic‐like connectivity between astrocytic output sites and PP receptive sites.

## Main Points

- Astrocyte signaling controls synaptic strength in a hippocampal memory circuit.
- The control is circuit‐specific and gated by the cytokine TNFα.
- Inflammation/infection over‐activates this mechanism causing synaptic alteration and reduced memory performance.

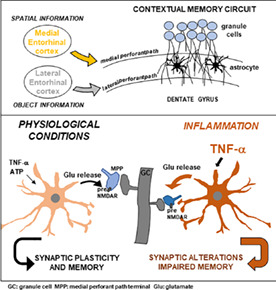

## 1 INTRODUCTION

Astrocytes are an abundant glial cell population, which tiles the entire central nervous system (CNS) and is thought to play essential roles in its function. However, still today, the exact roles of astrocytes are not completely understood. There are at least two different reasons for this: (a) Astrocytes are highly complex and unconventional cells, therefore methodologically challenging to study; (b) astrocyte studies were hindered for decades by conceptual views and experimental strategies dominating Neuroscience research. For example, electrophysiology, which brought in the 1950s to the exciting discovery of the neuronal electrical excitability and its key role in brain information processing, was somehow deleterious to the advance of astrocyte research, as it showed that astrocytes lack any electrical excitability, which was interpreted by many as astrocytes being passive cells *tout court*. However, progressive introduction of new experimental approaches such as molecular biology in the 80s, intracellular calcium (Ca^2+^) imaging in the 90s and cell‐specific mouse genetics in the beginning of the XXI Century, overturned this view and showed that astrocytes are chemically excitable, not passive, actively communicate with neurons and other brain cells, and contribute to shaping the physiology of synaptic circuits, and participate to their alterations in pathology.

In a first wave of studies, astrocytes were shown to sense the ongoing activity at neighboring synapses by means of neurotransmitter receptors expressed on their membranes and to respond to it via intracellular Ca^2+^ elevations and release of neuroactive molecules inducing synaptic modulation (reviewed in Haydon, 2001). These findings led to the new concepts of neuron‐astrocyte bidirectional communication (Bezzi et al., 1998; Pasti et al., 1997) and “tripartite synapses” (Araque et al., 1999), according to which astrocytes should be considered integral structural‐functional elements of the synapses. A second wave of studies expanded this view by showing that astrocytes contribute not only to local regulation of synaptic functions and plasticity, but also to the oscillatory patterns of larger neuronal networks, and thereby to shaping behavior, including learning and memory (reviewed in Santello et al., 2019).

In this review, we recapitulate and discuss work of the last 20 years by our group addressing the role, mechanisms, and implications of the bidirectional communication between neurons and astrocytes at the entorhinal cortex (EC)‐hippocampal dentate gyrus (DG) circuits involved in memory processing as well as in memory disturbances in Alzheimer's disease (AD) and other neurological conditions.

## 2 THE EC‐DG CIRCUITRY IN MEMORY AND MEMORY IMPAIRMENT

### 2.1 EC‐DG circuit organization and function

**FIGURE 1:**
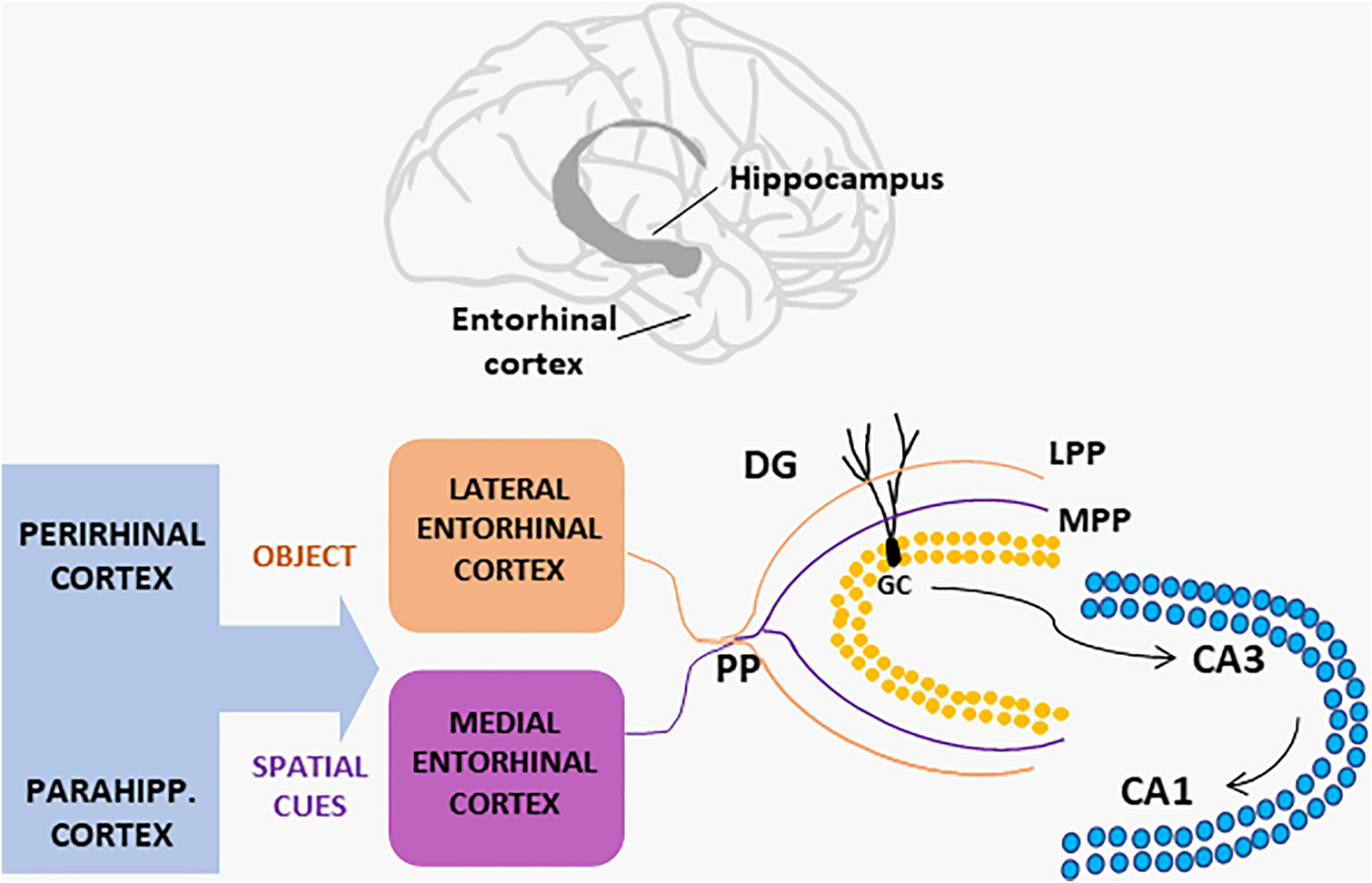
Scheme of the entorhinal cortex‐hippocampal connections. The hippocampal formation is located in the temporal lobe of the brain and receives contextual information from the entorhinal cortex via its projections (the perforant path, PP) to the dentate gyrus (DG). The medial entorhinal cortex conveys spatial information about the context received from the perirhinal cortex, whereas the lateral entorhinal cortex conveys sensory information about the nature of the context (the object) received from the parahippocampal cortex. PP axons segregate into the lateral and medial PP innervating respectively the outer third (OML) and middle third (MML) of the dentate molecular layer. Granule cells (GC) send projections to CA3 pyramidal neurons, which in turn project to the CA1 area

The DG is a major component of the hippocampal trisynaptic circuit, conveying information from the EC to the CA3, which in turn projects to the CA1 region, thereby participating in memory encoding, consolidation, and recall (Hainmueller & Bartos, 2020). Granule cells (GCs) are the major cell population present in the DG and receive polymodal sensory input from the parahippocampal and perirhinal cortices (Figure 1). The EC represents the relay station for these cortical inputs and projects to the hippocampus via the so called perforant path (PP) (Witter, 2007). PP axons originate mainly from layer II of the EC and can be divided in two distinct bundles, the medial and the lateral PP (MPP and LPP), based on the region of origin of the projections, that is, the medial or the lateral EC (MEC or LEC). Thus, LPP and MPP are anatomically segregated, and innervate respectively the most superficial third and the middle third of the hippocampal dentate molecular layer (HDML, Dolorfo & Amaral, 1998a; Witter & Moser, 2006) (Figure 1). The first retrograde labelling studies conducted in the 1990s demonstrated a topographical organization of the EC‐DG connections along the septo‐temporal axis of the hippocampus (Dolorfo & Amaral, 1998b). Noteworthy, the two sets of EC projections not only are anatomically segregated but also deliver inputs of different nature to the hippocampus, thus implying that MEC and LEC mediate different functions. LPP transmits olfactory, auditory, and visual information to the DG while MPP conveys spatial information. This functional divergence is supported by unit recording studies showing that MEC contains neurons with spatial firing properties, the so‐called grid cells (Hafting et al., 2005; Moser et al., 2017; Sargolini et al., 2006; Solstad et al., 2008), whereas neurons in LEC produce little spatial effects (Hargreaves et al., 2005; Yoganarasimha et al., 2011). The evidence for a differential contribution by the two pathways to learning and memory processes was reinforced by behavioral studies. In these studies, animals subjected to selective lesions in either MEC or LEC showed distinct types of behavioral impairment: MEC injury caused defects mainly in place learning paradigms, whereas LEC injury mainly in contextual learning ones (Burwell et al., 2004; Ferbinteanu et al., 1999; Hunsaker et al., 2007). At the level of the DG, visual object and spatial information are combined for a conjunctive encoding that supports formation of episodic memory in the hippocampus (Hargreaves et al., 2005; Hunsaker et al., 2007; Knierim et al., 2006). Another memory process involving the DG is “pattern separation”, whereby similar pieces of incoming information, such as those from two similar contexts, are distinguished via separation and representation by distinct (orthogonal) sets of neurons in the output network (Kohonen, 1984; Rolls, 1996). The sparse firing activity of dentate GCs and the larger size of the GC layer with respect to the EC‐PP input allow the divergence of information along the EC‐GC‐CA3 connections. Conclusive evidence for the involvement of the DG in memory formation came from experiments in which reactivation of memory‐specific neuronal ensembles, or engrams, in DG via optogenetic stimulation artificially reproduced memory recall of freezing behavior in mice (Liu et al., 2012; Ramirez et al., 2013).

### 2.2 Memory role of neurogenesis

Hippocampus‐mediated memory functions are influenced by the process of adult neurogenesis, which takes place at the level of the sub‐granular zone (SGZ) of the DG (for a review see Deng et al., 2010). The role of this process was revealed by studies in which DG neurogenesis was ablated via either X ray irradiation, DNA methylating agents (methylazoxymethanol acetate, MAM) or use of transgenic mice, resulting in defective trace memories formation (Shors et al., 2001), pattern separation (Clelland et al., 2009) and learning and memory processes (Deng et al., 2009). The opposite was observed in situations inducing increased DG adult neurogenesis, for example, when learning and memory tasks (Gould et al., 1999) were tested in mice exposed to an enriched environment (Kempermann et al., 1997) or to voluntary running (van Praag et al., 1999). Moreover, induction of genetic expansion of the population of adult‐born GCs improved pattern separation in rodents (Sahay et al., 2011). Intriguingly, integration of new granule cells into the DG circuit may not only promote the formation of new memories but also, via rescaling of preexisting connections, the forgetting of already established ones (Akers et al., 2014). Newborn GCs appear to be preferentially innervated by LEC inputs, although inputs from MEC would be also necessary for their proper integration in the DG synaptic circuit (Woods et al., 2018). Moreover, LEC and MEC inputs would lead these cells to exert opposite modulatory effects on the activity of mature GCs (Luna et al., 2019). A note of caution in interpreting the role of adult neurogenesis in hippocampal memory processing comes from human studies in which the existence of adult neurogenesis has been questioned and is currently debated (Moreno‐Jiménez et al., 2021; Sorrells et al., 2021). Some studies reported the presence of neurogenesis in hippocampus only during the first postnatal period (Sorrells et al., 2018), whereas others reported it also in the healthy adult brain, but with a drastic decrease in Alzheimer's subjects (Boldrini et al., 2018; Eriksson et al., 1998; Moreno‐Jiménez et al., 2019). A better understanding of the contribution of the newly generated neurons in humans is central to the development of new therapeutic strategies for Alzheimer's disease (AD) and other neurological conditions characterized by impaired hippocampal memory functions and cognition (Hyman et al., 1984).

### 2.3 The EC‐DG circuit in Alzheimer's disease

In AD, layer II EC neurons are selectively affected by early deposition of amyloid‐beta protein (Aβ), a phenomenon observed in both humans and AD transgenic mouse models carrying human familial AD mutations (reviewed in Götz et al., 2018). Interestingly, Aβ deposition appears to prevail in the outer versus the medial HDML, suggesting a higher vulnerability of LPP versus MPP to AD pathology (Gómez‐Isla et al., 1996; Khan et al., 2014; Reilly et al., 2003). Consequently, both EC projecting neurons and downstream synapses in the DG region innervated by their axons display molecular and functional alterations leading to neuronal hyper‐excitability and impaired plasticity (Jacobsen et al., 2006; Jiang et al., 2021; Marcantoni et al., 2014; Palop et al., 2007). Quite similar alterations at the charge of layer II EC neurons are seen in both humans and mice during normal aging (Smith et al., 2000; Yassa et al., 2010). The specific determinants for selective susceptibility of layer II EC neurons to AD pathology and senescence are still unclear, but they could depend on the interaction of several developmental, morphological, functional, and molecular factors characteristic of these cells (Stranahan & Mattson, 2010).

## 3 ASTROCYTES SENSE SYNAPTIC TRANSMISSION IN THE EC‐DG CIRCUIT

**FIGURE 2:**
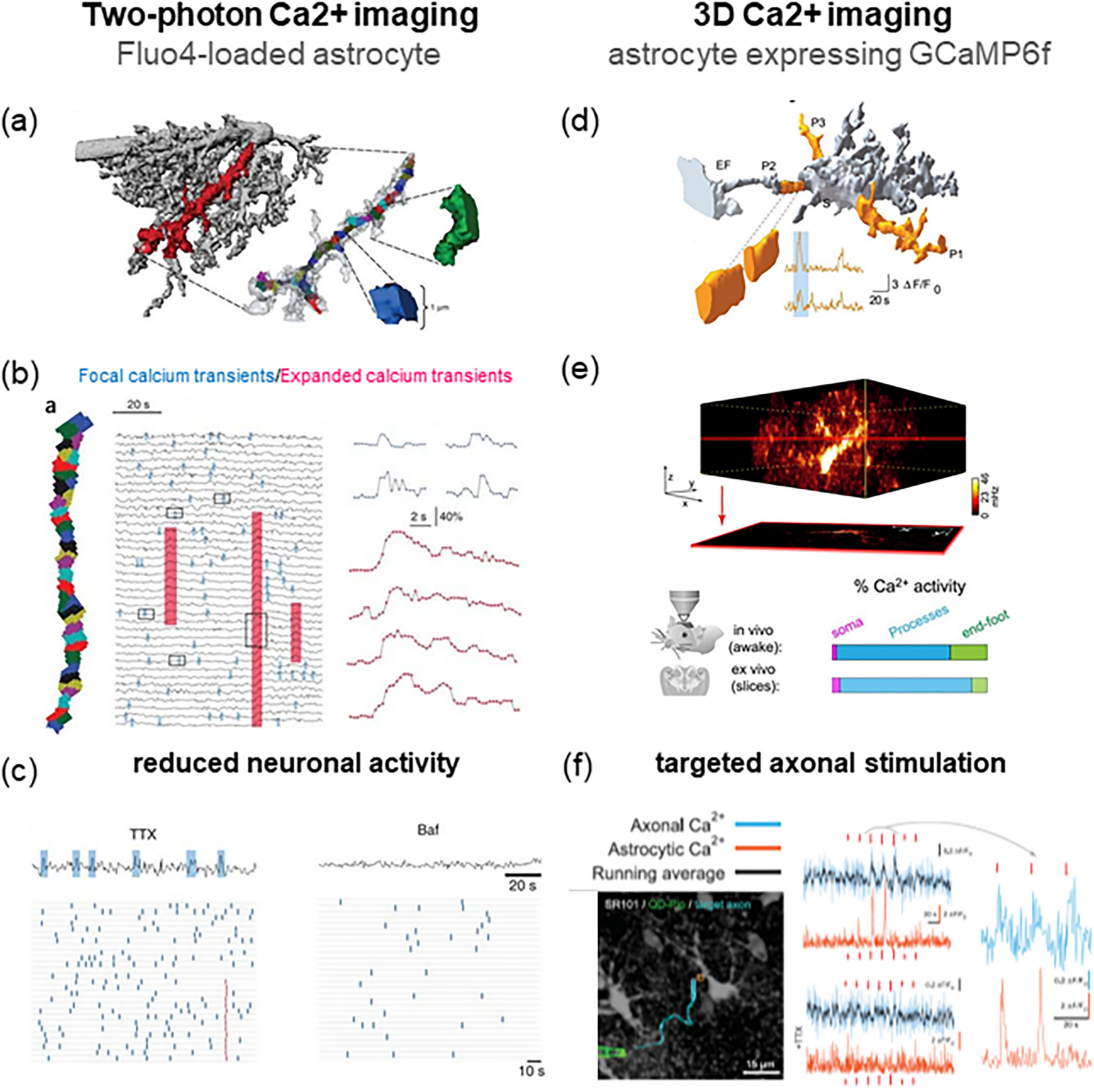
Innovative methods developed by our group to study Ca^2+^ dynamics in astrocytes and their dependency on neuronal activity. We monitored Ca^2+^ elevations in astrocytic processes by high‐resolution two‐photon Ca^2+^ imaging in the hippocampal dentate molecular layer (HDML) of the adult mouse. In the first study (Di Castro et al., 2011; left panels) we used the green Ca^2+^ indicator Fluo4 loaded into single astrocytes by the whole‐cell patch‐clamp technique. (a) Cell‐impermeant Texas red‐dextran was used to assess astrocyte morphology. An astrocytic process fully lying in the focal plane was selected for Ca^2+^ imaging and segmented in 1μm^2^ sub‐regions for Ca^2+^ analysis. (b) The calcium events observed in a process, were subdivided in two distinct groups: Focal events (small transients, limited to 1‐few sub‐regions, blue) and expanded events (high amplitude events, spanning several sub‐regions, red). (c) The block of action potential firing by TTX application reduced Ca^2+^ activity in astrocyte processes by affecting primarily expanded events. Bafilomycin A1(Baf), which depresses transmitter release by progressively depleting recycling vesicles of their transmitter content, abolished both focal and expanded Ca^2+^ transients in the astrocyte. In a second study (Bindocci et al., 2017; right panels) we developed a new method for 3D two‐photon Ca^2+^ imaging in astrocytes that selectively expressed the genetically encoded Ca^2+^ indicator (GECI) GCaMP6f. (d) Analysis of 3D Ca^2+^ dynamics was performed on segments (about 3 μm in diameter) of the reconstructed 3D core morphology of the astrocyte. (e) by rapidly scanning at least 30 individual focal planes, we could reconstruct endogenous Ca^2+^ dynamics in the entire volume of individual astrocytes, both in situ and in vivo in the awake, head‐fixed mouse. We found that most of the Ca^2+^ activity (in %) occurs in processes, is less frequent in end‐feet, and is very infrequent in the cell soma. (f) in dual color Ca^2+^ imaging experiments, we could show that HDML astrocytes respond to minimal stimulation of contiguous PP axons with time‐locked focal Ca^2+^ elevations (azur: Axonal Ca^2+^; orange: Astrocyte Ca^2+^) in a very small portion of their volume, and that TTX abolishes both axonal and astrocytic Ca^2+^ responses. The authors hold the copyright to reproduce panels (a), (b) and (c) Di Castro et al, Nature Neuroscience 2011 and Habbas et al., Cell 2015. Panels (d), (e) and (f) in Figure 2 are “Reprinted/adapted from Bindocci E, Savtchouk I, Liaudet N, Becker D, Carriero G, Volterra A. Three‐dimensional Ca^2+^ imaging advances understanding of astrocyte biology. Science. 2017 May 19; 356 (6339): eaai8185. doi: 10.1126/science.aai8185. © The authors, some rights reserved; exclusive licensee AAAS. Distributed under a CC BY‐NC 4.0 license http://creativecommons.org/licenses/by‐nc/4.0/

In the context of regulation of EC‐DG circuit function, work of several years from our lab has described an unconventional control exerted by HDML astrocytes on excitatory synapses onto GCs (PP‐GC synapses). We identified a glutamatergic input of the astrocytes onto PP fibers that increases their release probability (Di Castro et al., 2011; Jourdain et al., 2007; Santello et al., 2011; Savtchouk et al., 2019). Our initial key questions were centred on whether and how HDML astrocytes sense neuronal activity in the PP‐GC circuit: for example, what levels of neuronal activity induce Ca^2+^ elevations in astrocytes, and whether the type of astrocytic response changes in relation to the level of neuronal activity. We addressed these questions by using a combination of patch‐clamp electrophysiology and Ca^2+^ imaging in situ and in vivo. Our initial studies in hippocampal slices showed that repetitive stimulation of PP afferents (30 Hz) with a bipolar electrode positioned in the HDML consistently induced transient Ca^2+^ elevations in Fluo4‐loaded astrocytes. These astrocytic Ca^2+^ transients were abrogated upon perfusion of tetrodotoxin (TTX), a blocker of neuronal action potentials, demonstrating that HDML astrocytes respond to the firing of PP fibers (Jourdain et al., 2007). In these early studies, we monitored Ca^2+^ elevations in astrocytes at a relatively low‐resolution scale, as many other groups at the time, focusing mainly on cell body and main processes dynamics. In subsequent studies, we developed a more advanced two‐photon imaging approach that permitted us to monitor Ca^2+^ dynamics in the astrocytic processes with high temporal and spatial resolution (Di Castro et al., 2011, Figure 2). We discovered that HDML astrocytes of young adult mice display local Ca^2+^ transients confined to their processes in the absence of any external stimulation. These transients appear to represent distinct types of local responses to the endogenous neuronal activity present in the slices. We tentatively identified two main types: (a) “focal” Ca^2+^ events, very limited in space (one to few μm diameter) and time (sub‐second half‐time), and insensitive to TTX; and (b) “expanded” Ca^2+^ events, larger (several μm), longer‐lasting (about 3 s half‐time) and sensitive to TTX (Figure 2b, c). As for the origin of the small focal events, their TTX‐insensitivity indicates that they do not depend on action potential firing. However, they were inhibited by bafilomycin A1, which prevents acidification and refilling of synaptic vesicles and other acidic compartments (Zhou et al., 2000), and stimulated by local sucrose application, a treatment that triggers the immediate secretion of readily releasable synaptic vesicles (Rosenmund & Stevens, 1996; reviewed in Kaeser & Regehr, 2017). Based on the opposite modulation of focal events by the two above pharmacological treatments, both widely used in classic synaptic physiology studies to interfere with vesicle release, we tentatively concluded that focal events are evoked by spontaneous miniature synaptic events at PP‐GC synapses (Di Castro et al., 2011, see also Sun et al., 2014). Of course, more direct evidence in addition to pharmacological data is necessary for a conclusive demonstration of the synaptic origin of these events. As for expanded events, given their TTX‐dependency, we attributed their origin to the endogenous firing of PP fibers in our slices, mostly consisting of sparse action potentials (Di Castro et al., 2011). In keeping, we subsequently showed that minimal stimulation of PP axons causes time locked focal Ca^2+^ increases in both the stimulated axon and the spongy domain of a neighboring HDML astrocyte (Bindocci et al., 2017, Figure 2e). These data indicate that HDML astrocytes can sense individual action potentials, that is, the basal level of activity in the circuit. In line with this conclusion, a study performed in the CA1 region found that stratum radiatum astrocytes responded to minimal stimulation of Schaffer collateral axons with a time‐locked and spatially contiguous local Ca^2+^ elevation (Panatier et al., 2011). The capacity of astrocytes to sense basal synaptic activity was, however, disputed by another study performed in the CA3 region, in which multiple stimuli to mossy fiber axons were required to consistently observe large astrocyte Ca^2+^ responses extending to both processes and soma (Haustein et al., 2014). We think that this contrasting result may be explained by the different circuit studied (DG‐CA3), including possibly a different structural organization of synapses and astrocytes, and/or by the insufficient precision of the experimental design for detecting small astrocytic Ca^2+^ responses in proximity of the active axons (discussed in Bindocci et al., 2017).

The existence in astrocytes in situ of a class of small local Ca^2+^ events originally identified by us in the processes of HDML astrocytes based on their specific spatial–temporal characteristics (Di Castro et al., 2011) has been confirmed by many other studies in several brain regions. Such studies generally describe the signals as local and frequent “microdomains” of Ca^2+^ activity in astrocytic branchlets (e.g., Shigetomi et al., 2013). Importantly, using an innovative high‐resolution method studying Ca^2+^ dynamics in entire 3D astrocytes, we could confirm that astrocytic local Ca^2+^ activity exists not only in brain slices but also in vivo, in the head‐fixed awake mouse (Bindocci et al., 2017, Figure 2d). Moreover, this comprehensive approach allowed us to conclude that the local activity, which occurs asynchronously at myriads of locations in the processes and the spongiform domain (the “gliapil”), represents the most frequent type of Ca^2+^ activity of astrocytes (Bindocci et al., 2017). A recent study based on 3D‐STED microscopy in organotypic hippocampal slices provided evidence that the extremely intricate morphology of astrocytes in the neuropil provides the structural basis for compartmentalized intracellular Ca^2+^ signaling (Arizono et al., 2020). According to this study, local astrocytic Ca^2+^ signals occur in microdomains consisting of bulbous enlargements, named nodes that contact spines and are assembled along thin astrocytic “shaft” processes. Spatial and size correlations between astrocyte nodes and synaptic spines and boutons led the authors to propose that these structures represent the ultrastructural correlate of “tripartite synapses”.

The observation that synaptic activity triggers local Ca^2+^ elevations in HDML astrocytes is relevant to understanding the physiological role of such Ca^2+^ events. Identification of distinct types of Ca^2+^ elevation suggests that they could have different functional implications. Spontaneous, action potential‐independent, miniature synaptic release events are thought to maintain dendritic spines and stabilize their connections (McKinney et al., 1999; Sutton & Schuman, 2006). In this context, continuous focal signal exchanges between synapses and astrocytes might be also required to maintain in place established ‘tripartite’ connections and coordinate their plastic adaptations to different levels of activity (Bernardinelli et al., 2014). On the other hand, expanded Ca^2+^ events co‐ordinately activate several μm‐large domains of an astrocyte. This could be a condition required to convey enough regional Ca^2+^ excitation to defined glutamate release zones in the astrocyte (Bergersen et al., 2012; Bezzi et al., 2004) involving one or multiple contacts with synapses, and to synchronize the astrocytic modulatory input to all the connected synapses (see next Chapters for anatomical and mechanistic insight at PP‐GC synapses; Santello et al., 2011 for experimental support; Hamilton & Attwell, 2010 for a theoretical validation).

A pending question about the local Ca^2+^ activity in astrocytes is whether it is largely or just partly synaptically‐driven. A study in cortical slices favors the latter idea, based on the identification of a TTX‐ and bafilomycin A1‐independent Ca^2+^ activity in microdomains driven by Ca^2+^ release from mitochondria. Such activity would then be synaptic‐independent and intrinsic to the astrocytes, and would serve metabolic functions (Agarwal et al., 2017). How much of the overall local Ca^2+^ activity in an astrocyte is represented by synaptically‐driven versus nonsynaptically driven activity remains undefined, also because making a correct estimation of the relative proportions constitutes a methodological challenge (discussed in detail in Bindocci et al., 2017, text and supplementary material).

## 4 ASTROCYTES CONTROL FUNCTION AND PLASTICITY SELECTIVELY AT MPP‐GC SYNAPSES: PRESYNAPTIC TARGETING

The finding that astrocytes respond to synaptic activity with Ca^2+^ elevations, and apparently tune their responses to the level of the neuronal activity, calls for a next key question: does this Ca^2+^ response imply that astrocytes are involved in the control of synaptic functions and plasticity? We addressed this question by investigating the impact of stimulating or blocking astrocyte signaling on PP‐GC synaptic transmission. Experimental manipulation of the astrocytes produced functional changes of PP‐GC synaptic activity at multiple levels. In initial studies, we electrically stimulated HDML astrocytes and observed in response an increase in the frequency of the excitatory events recorded in GCs, both miniature (mEPSCs, in the presence of TTX) and spontaneous events (sEPSCs, without TTX). Moreover, electrical stimulation of astrocytes increased the amplitude of evoked synaptic events in GCs (evoked EPSCs, eEPSCs) and reduced their paired pulse ratio (PPR). All these effects can be ascribed to a presynaptic modification of the glutamate release probability of PP‐GC synapses (Jourdain et al., 2007, Figure 3c). In subsequent work, we used the opposite strategy, that is, we blocked astrocyte Ca^2+^ elevations. In these experiments, we performed double‐patch recordings of a GC and a neighboring HDML astrocyte and perfused the astrocyte with the Ca^2+^ chelator BAPTA added to the intracellular solution. When we minimally stimulated the PP to activate only few (Agarwal et al., 2017; Akers et al., 2014; Araque et al., 1999) fibers, we observed an increase in synaptic failures (without change in synaptic potency) selectively in the cell pairs where astrocytes were dialyzed with BAPTA (Di Castro et al., 2011, Figure 3d). Overall, these data indicate that astrocytes control presynaptic release probability at PP‐GC synapses. Moreover, they show that the astrocytic control is already in place during basal synaptic activity, in keeping with the observation that astrocytes respond with local Ca^2+^ elevation to low levels of neuronal activity (see Panatier et al., 2011 for similar findings at CA1 synapses). The astrocytic presynaptic control does not affect only synaptic transmission but also plasticity of PP‐GC synapses, notably long‐term potentiation (LTP). In this case, we recorded field excitatory postsynaptic potentials (fEPSPs) from DG molecular layer sites where astrocytes were patched with a BAPTA‐containing intracellular solution. We found that the magnitude of the LTP induced by high‐frequency stimulation (HFS‐LTP) of medial PP fibers was higher in fields with BAPTA‐loaded astrocytes than in fields with astrocytes not containing BAPTA (Savtchouk et al., 2019; Figure 4). This result is consistent with the idea that, under basal conditions, the astrocytic input contributes to the high release probability of PP‐GC synapses. When the astrocytic input is abolished by loading BAPTA into the astrocytes, the probability of neurotransmitter release at PP‐GC synapses is reduced (see effect on failure rate, Di Castro et al., 2011). Lower basal release probability is associated with an increased HFS‐LTP (Padamsey et al., 2017; Vyleta & Snyder, 2021). Overall, the presynaptic control exerted by HDML astrocytes on the basal release probability of PP‐GC synapses appears to set their level of prepotentiation thereby affecting the dynamic range for HFS‐LTP.

**FIGURE 3:**
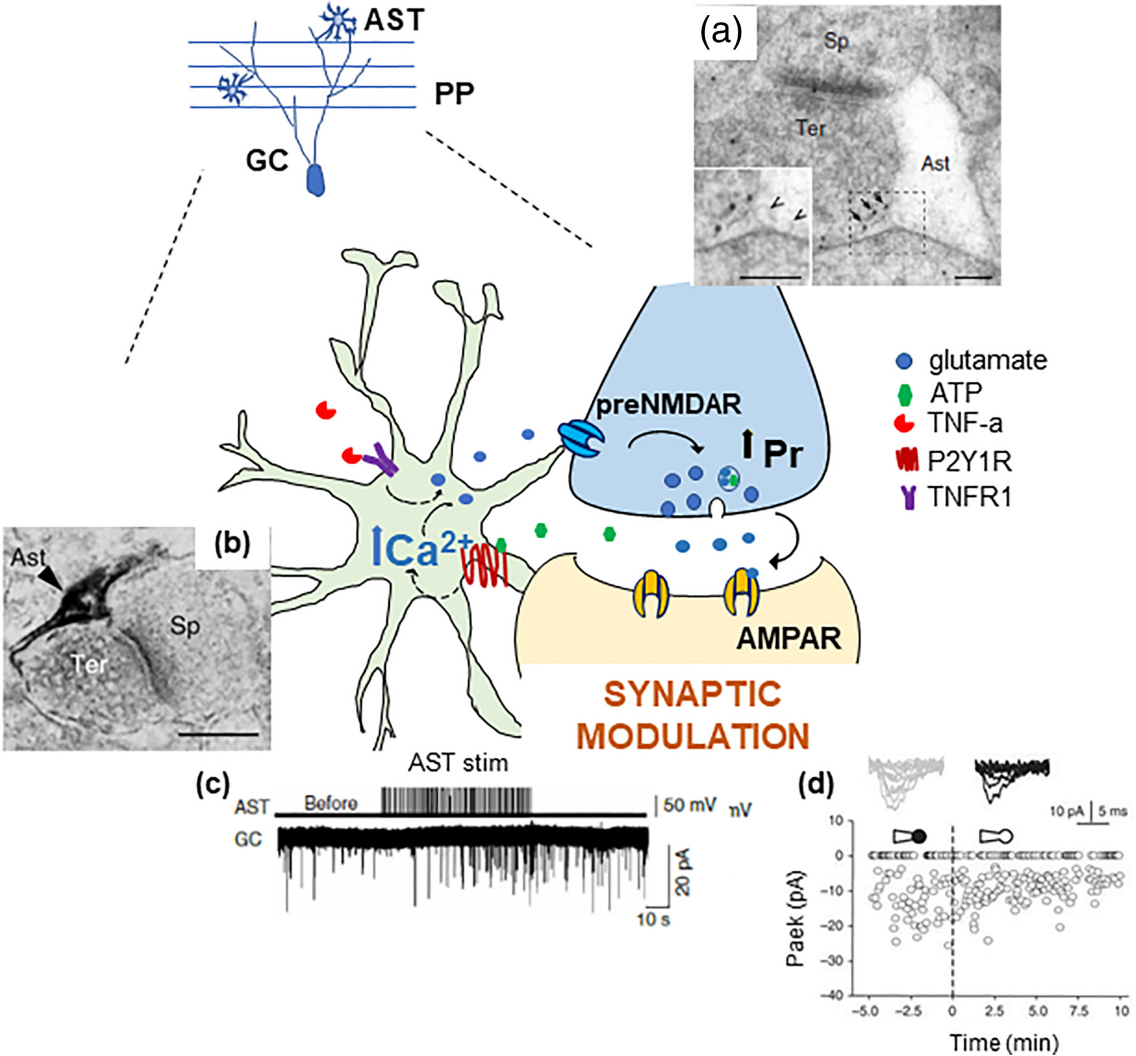
Molecular and functional evidence for a specific presynaptic astrocyte control on dentate perforant path‐granule cell synapses. Schematic representation of the pathways underlying the bidirectional communication between astrocytes (AST) and perforant path (PP) granule cell (GC) synapses in the dentate gyrus. The axonal terminals of PP fibers originating in the entorhinal cortex express pre‐synaptic NMDA receptors (preNMDAR) containing the GluN2B (electron microscopy image in [a]) and the GluN3A subunit (Savtchouk et al., 2019). Notice their extra‐synaptic location in PP terminals (arrows), facing astrocyte membrane displaying small clear vesicles (arrowheads). Intracellular Ca^2+^ elevations in astrocytes are induced by purinergic P2Y1R stimulation (P2Y1R immunoreactivity selectively in a perisynaptic astrocytic process at PP‐GC synapses is shown in the electron micrograph in [b]). Such elevations cause glutamate release onto pre‐NMDARs facing the astrocytic membranes. This event increases the probability of synaptic release (Pr) and results in the positive modulation of synaptic activity in GCs. We revealed the astrocyte modulation in several ways, for example, by either astrocytic electrical stimulation ([c], increased mEPSC frequency) or by perfusion of astrocytes with a BAPTA‐containing intracellular solution via a whole‐cell pipette ([d], increased synaptic failures). Endogenous ATP, possibly co‐released from glutamatergic terminals expressing the ATP vesicular transporter, VNUT (Larsson et al., 2012), could be the stimulus activating the astrocyte modulatory effect. Constitutive TNFα, via TNF receptor type 1, gates the glutamate release process from the astrocyte (Santello et al., 2011). Excerpt from Jourdain et al., 2007 (a, b and c) and Di Castro et al., 2011 (d). The authors hold the copyright to reproduce panels from Jourdain et al., Nature Neuroscience 2007 and Di Castro et al., 2011

**FIGURE 4:**
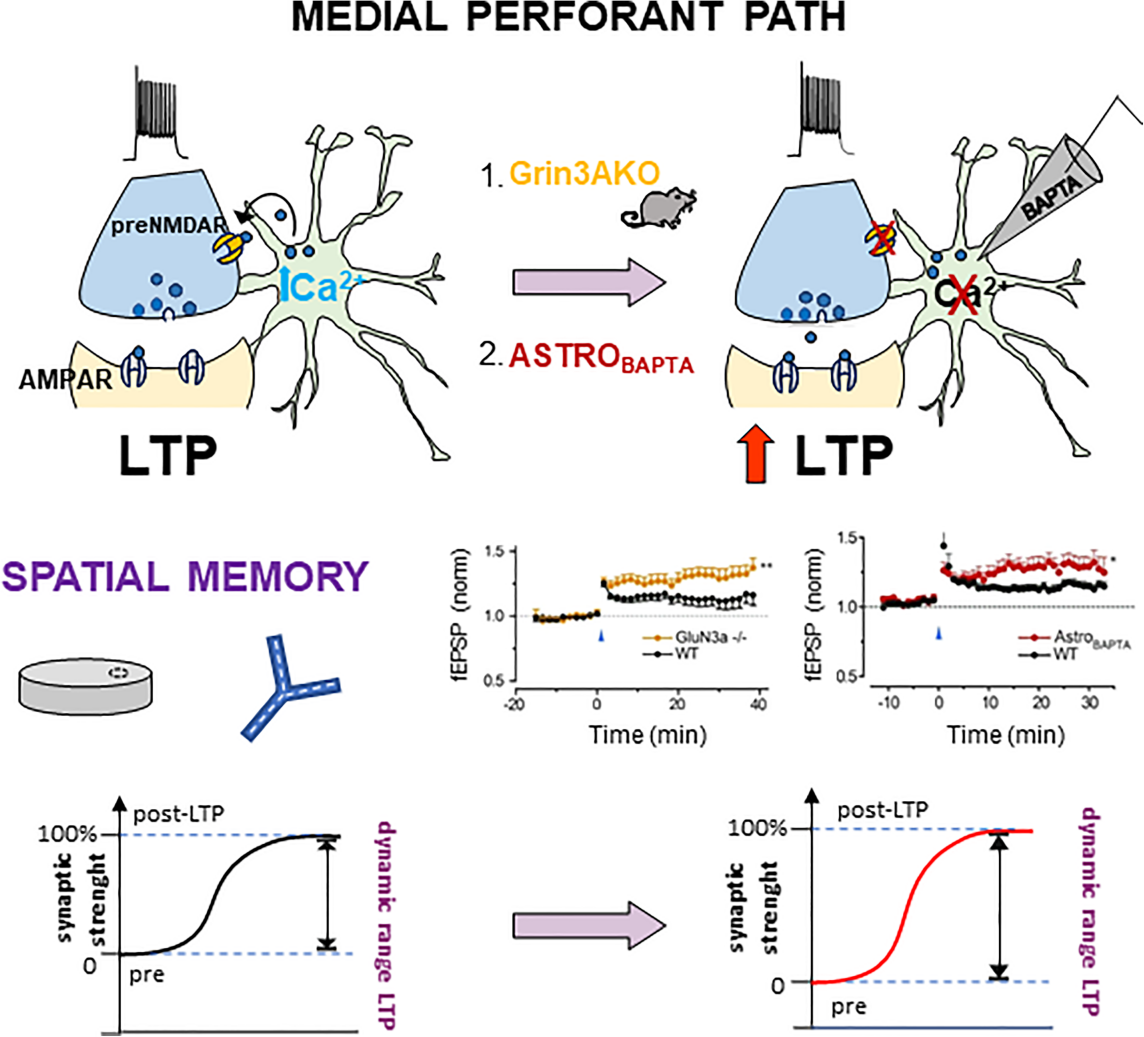
Circuit‐specific astrocytic control of LTP onto medial perforant path fibers via activation of presynaptic GluN3A‐NMDA receptors. Schematic illustration of our data showing that astrocytic signaling onto GluN3A‐containing pre‐NMDA receptors controls the dynamic range for LTP at MPP‐GC synapses by modulating the probability of glutamate release (Pr). Left panel: In the presence of intact astrocyte signaling, high‐frequency stimulation (HFS) of MPP fibers induces LTP and conveys spatial information cues to the DG. Right panel: When the astrocyte control is abolished by either genetic deletion of the GluN3A subunit (Agarwal et al., 2017) or by infusing BAPTA into the astrocyte (Akers et al., 2014), LTP magnitude is increased following HFS of MPP fibers, revealing the role of the astrocyte cascade. Based on these data, we propose (graphs in the lower part of the panels) that the astrocyte control sets the basal strength of MPP‐GC synapses to a significant level of pre‐potentiation, which leaves a range for further potentiation by HFS stimulation (left), that is smaller than the one seen when the astrocyte control on basal release probability is abolished by the above manipulations (right). Excerpt from Savtchouk et al., 2019. The authors hold the copyright under the CC BY‐NC‐ND license to reproduce figures from Savtchouk et al., PNAS 2019, https://creativecommons.org/licenses/by‐nc‐nd/4.0/ for the summary of terms and conditions

Expression of LTP at PP‐GC synapses had been previously associated with differences in the MK801 blocking rate between LPP and MPP, indicating a presynaptic modification of neurotransmitter release at MPP‐GC synapses (Min et al., 1998). As we will discuss in more detail in the next Chapters, our data are consistent with those initial observations. Notably, in a recent study, we demonstrated that the identified astrocytic control of release probability and HFS‐LTP is circuit‐selective, that is, occurs at MPP‐GC synapses but not at LPP‐GC synapses, and this because it involves activation of a peculiar type of presynaptic NMDAR (preNMDAR) that is present in MPP terminals but not in LPP terminals (Savtchouk et al., 2019, see also next Chapter). Intriguingly, such receptors can be activated even at high Mg^2+^ concentration (2 mM), suggesting that they have low voltage‐dependent Mg^2+^ block. The receptors contain the GluN2B subunit, as indicated by both immunogold electron microscopy (EM) evidence (Jourdain et al., 2007) and by the pharmacological effect of ifenprodil, a GluN2B‐specific antagonist, which reduces eEPSCs (and increases PPR) selectively at MPP‐GC synapses (Savtchouk et al., 2019). However, the low Mg^2+^ sensitivity of preNMDARs cannot be explained by the presence of the GluN2B subunit and implies that these receptors incorporate other subunits (Paoletti et al., 2013). Indeed, by immunogold EM, we found expression in MPP but not LPP synaptic terminals of GluN3A, an NMDAR subunit that usually reduces Ca^2+^ conductance and Mg^2+^ sensitivity of the receptors (Henson et al., 2010). In the terminals, GluN3A and GluN2B have analogous extra‐synaptic localization, facing astrocytes (Jourdain et al., 2007; Savtchouk et al., 2019). Consistent with the above observations, genetic ablation of the GluN3A subunit mimicked and occluded the functional effects of ifenprodil at MPP‐GC synapses, as well as increased their dynamic range for HFS‐LTP, an effect analogous to the one produced by blocking astrocyte signaling with BAPTA (Savtchouk et al., 2019, Figure 4).

Prior electrophysiological and pharmacological studies revealed that MPP and LPP inputs to GCs have distinctive features, including different functional properties, modulation, and drug selectivity. For example, LPP‐evoked responses are sensitive to group III mGluR agonists (Macek et al., 1996), whereas MPP‐evoked responses are sensitive to carbachol and group II mGluR agonists (Christie & Abraham, 1992a, 1992b; Colino & Malenka, 1993; Froc et al., 2003; Kahle & Cotman, 1989; McNaughton, 1980; McNaughton & Barnes, 1977). Moreover, MPP and LPP inputs respond differently to electrophysiological paired‐pulse protocols, usually resulting in paired pulse facilitation (PPF) when LPP is stimulated, and in paired pulse depression (PPD) when MPP is stimulated. This indicates that MPP inputs have higher release probability than LPP inputs (McNaughton, 1980; Min et al., 1998). Also, the induction of LTP shows differences in the two pathways and is more successful when MPP is stimulated rather than LPP (Colino & Malenka, 1993). This may depend on different mechanisms and receptors mediating the cellular processes underlying LTP in the two pathways. At LPP fibers, LTP expression would involve activation of postsynaptic mGlu5 receptors, followed by production of the endocannabinoid 2‐arachidonoyl‐sn‐glycerol (2‐AG), which acts retrogradely to increase glutamate release from the presynaptic site (Wang et al., 2016). In keeping, manipulations of endocannabinoid signaling, either suppressing or enhancing LTP, produce corresponding effects on LPP‐dependent learning such as odor discrimination (Wang et al., 2016). The mechanistic basis for LTP expression in MPP is less understood, except for the reported presynaptic basis and the involvement of NMDA receptors (Min et al., 1998). Our findings show that astrocytes exert a circuit‐specific control of MEC inputs via GluN3A‐containing preNMDAR in MPP terminals and agree with a key role of preNMDARs in controlling synaptic strength in the MEC‐DG circuit. Genetic deletion of GluN3A likely removes the astrocyte‐induced prepotentiation and allows for stronger LTP of MPP‐GC synapses. This circuit‐specific enhancement of LTP could be the substrate for the higher recognition and spatial memory performances observed in GluN3A‐KO mice (Mohamad et al., 2013).

As discussed in Chapter 1, MEC and LEC projections convey different kinds of information to the DG and contribute differently to hippocampal learning and memory processes. Our results add a new circuit‐specific control mechanism, astrocyte‐dependent, which contributes to make MPP‐GC and LPP‐GC synapses functionally distinct. By this mechanism, astrocytes may directly contribute to the control of MEC‐driven behaviors (see Discussion).

## 5 MECHANISM OF THE ASTROCYTE CONTROL OF MPP‐GC SYNAPSES: ROLE OF P2Y1 RECEPTORS, VESICULAR GLUTAMATE RELEASE AND GluN3A‐CONTAINING pre‐NMDAR

If PP‐GC synaptic activity induces Ca^2+^ elevations in HDML astrocytes, and this in turn results in modulation of MPP‐GC synapses, astrocytes must possess defined intracellular signaling systems capable of translating the inputs into outputs via Ca^2+^ encoding. Several receptors have been involved in the astrocytic detection of synaptic activity in hippocampus, including metabotropic glutamate, muscarinic acetylcholine, endocannabinoid CB1, GABA_B_ and purinergic P2Y receptors, all G protein‐coupled receptors (GPCRs) that transduce extracellular signals into Ca^2+^ elevations (reviewed in Kofuji & Araque, 2021). At PP‐GC synapses, we demonstrated that at least part of the synaptically‐evoked astrocytic Ca^2+^ elevations are mediated by purinergic P2Y1 receptors (P2Y1R), prominently expressed in astrocytic processes surrounding the excitatory synapses (Jourdain et al., 2007; Figure 3b). Thus, perfusion of the selective P2Y1R antagonist, adenosine‐3‐phosphate‐5‐phosphosulfate (A3P5PS), significantly reduced both Ca^2+^ elevations evoked in astrocytes by electrical stimulation of PP afferents and spontaneous astrocytic Ca^2+^ elevations, triggered by the endogenous PP firing in the slices. In parallel, the drug reduced the amplitude of eEPSCs in GCs increasing their PPR, and decreased the frequency of sEPSCs (Jourdain et al., 2007). Consistently, in minimal stimulation experiments, MRS2179, another potent P2Y1R antagonist, increased synaptic failures (Di Castro et al., 2011). MRS2179, however, had no effect on mEPSC events recorded in the presence of TTX (Santello et al., 2011), while the P2Y1R agonist, 2MeSADP, increased their frequency (Jourdain et al., 2007; Santello et al., 2011). Taken together, these data indicate that activation of astrocyte P2Y1R‐dependent signaling occurs in response to action‐potential‐dependent activity of PP‐GC synapses, but not following miniature synaptic events. A pertinent question concerns the endogenous source of the ATP that activates astrocyte P2Y1Rs, as PP‐GC synapses are known to release glutamate. Intriguingly, immunogold studies reported the presence in PP terminals not only of vesicles expressing VGLUT1, a vesicular glutamate transporter, but also of vesicles expressing VNUT, an ATP vesicular transporter (Larsson et al., 2012). Therefore, ATP could be released during activity of PP‐GC synapses, as described at other glutamatergic hippocampal synapses (Pankratov et al., 2006).

To explore the GPCR‐ and Ca^2+^‐dependent mechanism leading to astrocyte‐mediated synaptic modulation, we used total internal reflection microscopy (TIRF), for the first time in astrocyte research. These studies showed that activation of GPCRs in cultured astrocytes, including P2Y1Rs, induces Ca^2+^‐dependent exocytosis of VGLUT‐expressing vesicles, whose transmitter release was revealed by time‐locked activation of NMDARs expressed on co‐cultured sniffer cells (Bezzi et al., 2004; Domercq et al., 2006). These observations were confirmed also by other labs (Bowser & Khakh, 2007). We supplemented this direct mechanistic evidence in vitro with three congruent indirect lines of evidence in situ. The first one consisted in postembedding immunogold EM experiments, by which we identified the presence in HDML astrocytes of small and clear vesicular organelles (synaptic‐like microvesicles, SLMVs) co‐expressing VGLUTs (mainly VGLUT1), VAMP SNARE proteins and L‐glutamate (Bergersen et al., 2012; Bezzi et al., 2004; Domercq et al., 2006). The astrocytic SLMVs were often seen at sites near the plasma membrane directly in front of sites in the extra‐synaptic portion of the PP terminals that expressed the NMDA receptor subunit GluN2B (Jourdain et al., 2007, Figure 2a). The second line consisted in single‐cell RT‐PCR experiments, by which we confirmed the presence of VGLUT1 and VGLUT2 transcripts in HDML astrocytes. Importantly, both EM and PCR experiments were performed in specimens from the adult brain and their results indicated that the molecular determinants for glutamate exocytosis were expressed in just part (20%–30%) of the HDML astrocytes (Jourdain et al., 2007, see also Discussion). A third line consisted in dual patch‐clamp recordings in pairs of an HDML astrocyte and a GC. In control experiments, electrical stimulation of the astrocyte induced synaptic potentiation in about 30% of the paired GCs. This effect was, however, abolished in experiments in which the astrocyte was internally perfused with tetanus neurotoxin's light chain (TeNT_LC_), a blocker of vesicular exocytosis that acts specifically on VAMP2 and VAMP3. In contrast, the effect persisted in experiments in which the astrocyte was perfused with the inactive toxin mutant, TeNT_LCE271A_.

Concerning preNMDARs, the molecular target of astrocyte‐released glutamate at MPP‐GC synapses, our studies comprising pharmacological inhibition, genetic deletion, and EM immunogold experiments, let us conclude that they are composed of GluN1‐N2B‐N3A subunits (Jourdain et al., 2007; Savtchouk et al., 2019, see also Chapter 3). Noteworthy, a study that utilized similar experimental approaches, came to the same conclusions about the existence of GluN1‐N2B‐N3A preNMDARs in the juvenile visual cortex, acting to enhance neurotransmitter release and mediate spike timing‐dependent synaptic plasticity (Pérez‐Otaño et al., 2016). However, visual cortex preNMDAR were reported to function as autoreceptors, which is not the case at MPP‐GC synapses, where the receptors are not activated by spontaneous glutamate release from MPP terminals. Indeed, blocking the receptors with D‐APV, a broad‐spectrum NMDAR antagonist, did not produce any effect on mEPSCs frequency (Savtchouk et al., 2019). In contrast, blocking NMDARs, in this case with ifenprodil, produced a large presynaptic inhibitory effect on both sEPSCs and eEPSCs, in all analogous to the effect of P2Y1R antagonists (Jourdain et al., 2007), indicating that preNMDARs become active under conditions of enhanced glutamate release. Possible mechanisms underlying their activation include synaptic spillover or release from the astrocytes. The high density of glutamate transporters on the perisynaptic astrocytic membrane, assuring efficient uptake of synaptic glutamate (Santello et al., 2011), and the close apposition between preNMDARs and astrocytic membranes (Jourdain et al., 2007; Savtchouk et al., 2019) support an astrocytic origin of the released glutamate.

Taken together, the above data provide, in our opinion, significant evidence that subpopulations of HDML astrocytes are activated during physiological transmission at PP‐GC synapses via P2Y1R and control synaptic function by releasing neuroactive glutamate via a vesicular mechanism acting onto atypical preNMDARs on MPP terminals. We admit that our data do not reach conclusive demonstration of the glutamatergic gliotransmission pathway, in that they do not directly demonstrate vesicular exocytosis of glutamate from astrocytes in situ and its causal role in the induction of the observed synaptic effects (criticism raised in Hamilton & Attwell, 2010). On the other hand, we think that more general criticisms questioning the existence of glutamatergic gliotransmission in the adult brain (Barres, 2008; Fiacco et al., 2009; Fiacco & McCarthy, 2018; Nedergaard & Verkhratsky, 2012) underestimate the ensemble of the above experimental results (see also Discussion).

## 6 THE CYTOKINE TNFALPHA CONTROLS THE ASTROCYTE INPUT TO PP‐GC SYNAPSES: ALTERED COGNITIVE PROCESSING IN PATHOLOGY

A surprising discovery about the GPCR‐dependent signal‐transduction in HDML astrocytes is that it involves an additional intermediate step of regulation, set by the levels of the cytokine tumor necrosis factor‐alpha (TNFα, Bezzi et al., 2001; Domercq et al., 2006; Santello et al., 2011). Our initial discovery of the role of TNFα in astrocytes (Bezzi et al., 2001), came at about the same time when TNFα was shown to control trafficking of AMPA receptors at excitatory neuronal synapses (Beattie et al., 2002) and we will see that there are several important similarities in the two actions of the cytokine. The TNFα‐dependent AMPAR control occurs physiologically at very low (constitutive) levels of the cytokine and participates in synaptic scaling (Stellwagen & Malenka, 2006), a phenomenon of homeostatic plasticity by which the strength of all synapses in a neuron is homogeneously reset by up or down scaling, depending on the previous activity history (Turrigiano, 2008). Mechanistically, TNFα induces up‐scaling of excitatory synaptic strength by promoting insertion of new AMPA receptors in the postsynaptic membrane. TNFα would act similarly at inhibitory synapses, where it promotes insertion of new GABA receptors in response to reduced synaptic activity (Stellwagen et al., 2005). Effects at both excitatory and inhibitory synapses are mediated by TNFα of glial origin acting on neuronal TNF receptor type 1 (TNFR1). The TNFR1 downstream signaling would facilitate exocytosis of the receptor subunits contained in transport organelles, a process sensitive to TeNT_LC_ for the membrane insertion of GluR1‐containing AMPA receptors (Lin et al., 2009).

In HDML astrocytes, constitutive TNFα functions as an obligatory factor for the induction of synaptically effective P2Y1R‐dependent gliotransmission at PC‐GC synapses. Specifically, TNFα controls glutamate exocytosis from the astrocytes (Santello et al., 2011; Santello & Volterra, 2012). P2Y1R activation with the receptor agonist, 2MeSADP, leads to an increased mEPSC frequency in GCs. However, we found that the 2MeSADP modulatory effect was abolished in slices from *Tnf−/−* mice or in slices from wild‐type mice incubated with sTNFR, a scavenger of the endogenous TNFα present in the slices, which revealed the essential control exerted by this cytokine. Importantly, addition of very low TNFα concentrations (60–150 pM) to *Tnf−/−* slices, fully rescued the synaptic effect of 2MeSADP. We then looked for the mechanism of the TNFα action in astrocytes. We first excluded a significant effect of the cytokine on the P2Y1R signaling leading to Ca^2+^ elevation. Therefore, we focused attention on the downstream glutamate release process. We performed TIRF experiments in cultured astrocytes using a specific fluorescent indicator of glutamate vesicle exocytosis, VGLUT1‐pHluorin, and evoked exocytosis by P2Y1R stimulation with 2MeSADP. P2Y1R‐induced glutamate exocytic events in *Tnf−/−* astrocyte cultures were identical in number to those in wild‐type cultures, but occurred in a desynchronized manner, spreading over a much longer interval. We could ascribe this slowdown of the release kinetics to a defect in docking of the vesicles at the plasma membrane in the absence of TNFα (Santello et al., 2011). The evidence in astrocyte cultures was reinforced by experiments in situ where the efficacy of P2Y1R‐dependent gliotransmission on mEPSCs frequency, absent in *Tnf−/−* slices, was rescued by the addition of DL‐threo‐beta‐benzyloxyaspartate (TBOA), a glutamate uptake blocker. Taken together, these data suggest that, in the absence of TNFα, glutamate is released slowly from HDML astrocytes and cannot reach sufficient extracellular concentration to activate neighboring preNMDARs because it is rapidly captured by the competing uptake. However, when uptake is inhibited with TBOA, despite the slow release, glutamate is now able to progressively accumulate extracellularly and eventually reaches the concentration required to activate pre‐NMDAR and mediate the synaptic effect of 2MeSADP.

The surprising finding that TNFα at constitutive (low picomolar) levels exerts a permissive control on the astrocyte regulation of synaptic activity, raised the next key question: what happens to this astrocyte pathway in conditions when TNFα levels increase, for example, under inflammatory or infective processes in the brain? Our results show that TNFα changes its role and directly triggers glutamate release from astrocytes in a dose‐dependent manner. Consequently, the excessive release of TNFα causes synaptic alterations, behavioral impairments and neurotoxic effects that are probably part of the neuropathology underlying human conditions as different as AIDS dementia and multiple sclerosis (Bezzi et al., 2001; Habbas et al., 2015, Figure 5). As first point, we documented the dose‐dependency of the TNFα‐induced astrocyte glutamate release. In cultured astrocytes, the cytokine starts to induce glutamate release at 300 pM, a few‐fold higher concentration than the one supporting P2Y1R‐dependent gliotransmission, and reaches its plateau effect at around 1.8 nM, a ten‐of‐folds higher concentration than its constitutive level (Bezzi et al., 2001; Santello et al., 2011). As second point, we identified a specific pathology‐related mechanism responsible for increased TNFα levels and noxious astrocyte glutamate release, activated by the human immunodeficiency virus (HIV) when it enters the brain (Bezzi et al., 2001). The mechanism involves excessive stimulation by the HIV envelope glycoprotein, gp120, of CXCR4, a GPCR chemokine receptor that is normally activated by the endogenous chemokine, stromal‐derived factor 1 (SDF‐1 or CXCL12). CXCR4 in the CNS is present mainly in microglia and its signal‐transduction involves release of TNFα. Some of the gp120 isoforms possess very high affinity for this receptor and are likely to activate it during HIV brain pathology, particularly at foci of reaction to the virus where activated astrocytes and microglia accumulate, causing massive increase in the local TNFα concentration. We showed in a cell culture model that gp120‐evoked TNFα release, mostly from reactive microglia, is sufficient to cause glutamate release from astrocytes by activating astrocyte TNFR1 signal‐transduction, and that the latter induces slow apoptotic death of neurons. We also reported that the same apoptotic neuronal death occurs in vivo upon gp120 injection in the brain and can be rescued by blocking the astrocyte signaling cascade (Bezzi et al., 2001). Therefore, this mechanism could contribute to the AIDS neurocognitive disorder, a brain pathology that in the past, before the introduction of the antiretroviral therapy, often evolved into frank dementia, and still today, despite the therapy, produces cognitive deficits in up to 50% of the subjects infected with HIV (Saylor et al., 2016). Noteworthy, this mechanism was at the time of our discovery among the first examples of neurodegeneration secondary to a glial alteration. This noxious interplay between microglia and astrocytes orchestrated by TNFα was later reported to be central also to other neurodegenerative disorders (Liddelow et al., 2017, Nature, see below).

**FIGURE 5:**
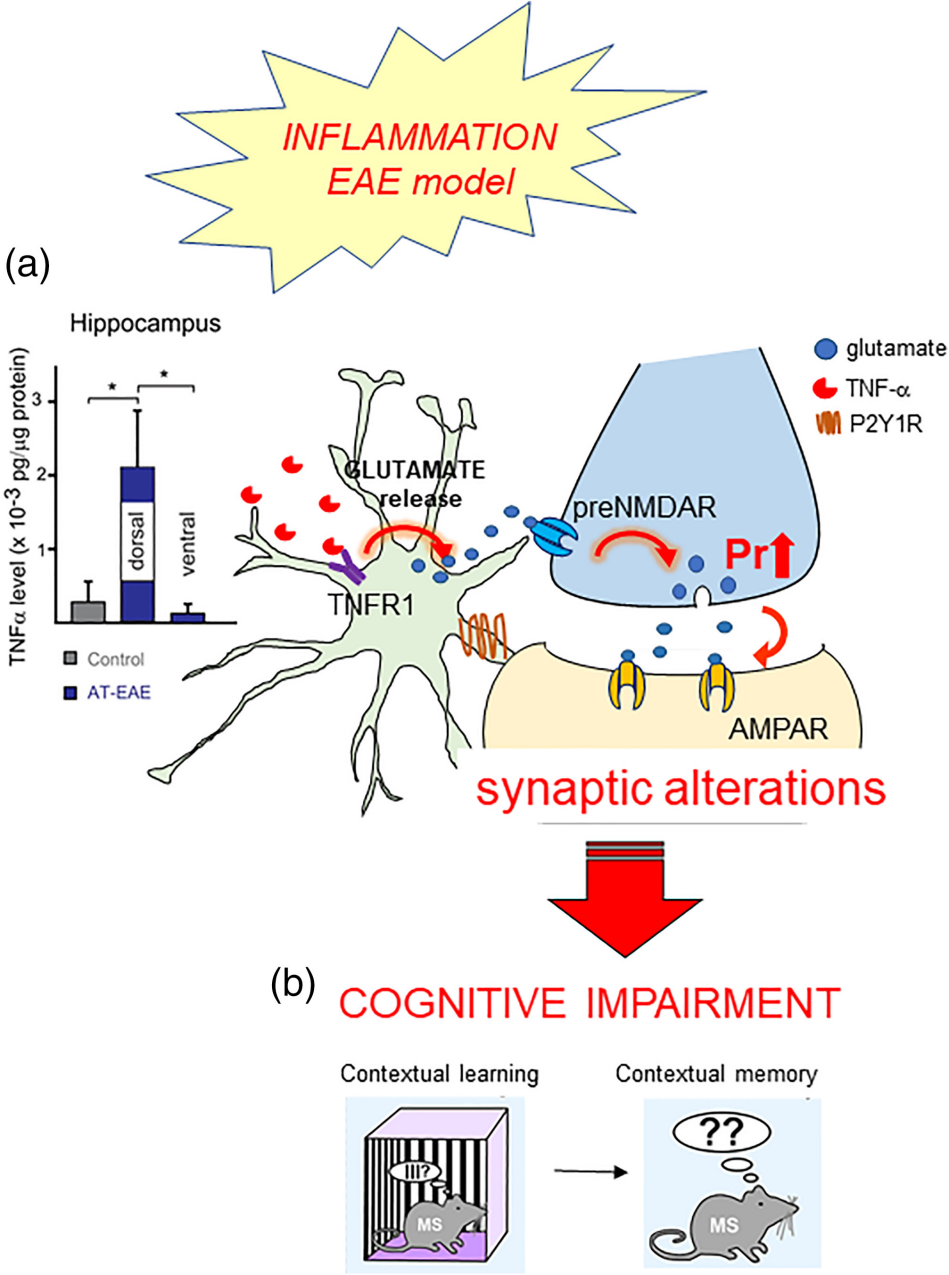
Pathophysiology and behavioral consequences of altered astrocyte‐neuron communication at PP‐GC synapses. Local leukocyte infiltration and tissue inflammation as in the course of multiple sclerosis (EAE mouse model) elevates TNFα levels selectively in the dorsal hippocampus (a). In turn, the cytokine, by stimulating TNFR1 on the astrocytes, triggers excessive astrocyte glutamate release, which results in alterations of PP‐GC synaptic transmission, ultimately responsible for contextual fear memory impairment ([b], details in Habbas et al., 2015). The authors hold the copyright to reproduce panels 5 from Habbas et al., Cell 2015

A second example of pathological alterations induced by enhanced TNFα levels via the astrocyte pathway comes from our studies in a murine model of multiple sclerosis, the experimental autoimmune encephalitis (EAE) model. In this model, local infiltration of leukocytes and brain inflammation sustained by reactive microglia occur in the dorsal hippocampus, notably in the DG near the third ventricle (Habbas et al., 2015, Figure 5). We measured in this region an 8‐fold increase in the tissue levels of TNFα, accompanied by persistent alteration of PP‐GC excitatory neurotransmission, which we showed to depend on both astrocyte TNFR1 and ifenprodil‐sensitive presynaptic NMDARs (Figure 5a). The ensemble of the synaptic alterations that we observed, which include increased mEPSC frequency, reduced PPR and reduced input/output curve for eEPSCs, suggests that the basal strength of PP‐GC synapses in EAE mice is set to an abnormally high level, which reduces the dynamic range for LTP (Habbas et al., 2015). In keeping, EAE mice do not perform well in the contextual fear conditioning test, which requires correct information processing in the EC‐DG circuit (see Chapter 1) and show memory deficits (Figure 5b). To better define the underlying mechanism, we developed a new transgenic mouse line carrying a floxed neo cassette in the TNFR1 coding sequence and an astrocyte‐specific inducible promoter. These mice are ubiquitous TNFR1KO that, upon tamoxifen injection, re‐express TNFR1 solely in astrocytes (Habbas et al., 2015). Thanks to this model, we conclusively demonstrated that the synaptic and cognitive behavioral alterations observed in EAE mice depend on anomalous astrocyte TNFR1‐dependent signaling. Thus, these alterations, absent when EAE was induced under ubiquitous TNFR1KO, were reinstated by just re‐expressing TNFR1 in astrocytes (Habbas et al., 2015). Importantly, while multiple sclerosis is mainly considered a white matter pathology, increasing evidence shows that it involves also gray matter alterations, and up to 50% of the patients suffer cognitive symptoms whose neurobiological basis has remained unclear so far (Chiaravalloti & DeLuca, 2008). The TNFα‐dependent pathway via the astrocytes that we described provides a mechanistic link between the presence of an inflammatory/infiltration locus in the DG and the insurgence of functional alterations of the synaptic circuitry in that region, which result in a reduced cognitive performance.

Several neuropsychiatric conditions have been reported to be associated to increased CNS levels of TNFα, suggesting that the above mechanism could operate in a wide range of brain pathologies to link inflammation to cognitive impairment. Indeed, cognitive symptoms are seen in CNS inflammatory states due to bacterial or viral infections, or even in peripheral inflammatory states resulting in septic encephalopathy (Clark et al., 2010; Swardfager & Black, 2013). In AD, TNFα signaling via TNFR1 has been implicated in learning and memory deficits (He et al., 2007), although a specific role for the astrocyte TNFR1 receptor has not been identified yet. In the hippocampus, deposition of Aβ plaques surrounded by local inflammation involving astrocytes and microglia, causes circuit unbalances and enhanced neuronal excitability (Palop et al., 2007). Astrocytes surrounding Aβ deposits were found to be hyperactive, displayed altered Ca^2+^ signaling (Delekate et al., 2014; Kuchibhotla et al., 2009) and increased gliotransmission (Gómez‐Gonzalo et al., 2017). In an AD transgenic mouse model, the astrocytic Ca^2+^ hyperactivity was sensitive to P2Y1R blockade in vivo (Delekate et al., 2014), suggesting that the gliotransmission pathway we identified could be involved in AD‐induced circuit alterations and memory deficits. Moreover, TNFα and a few other factors released by reactive microglia have been shown to drive deleterious phenotypic transformation of astrocytes, predisposing them to be neurotoxic (Liddelow et al., 2017). Markers of such phenotypic switch of the astrocytes have been found in postmortem tissues from patients of various neurological conditions, including multiple sclerosis, amyotrophic lateral sclerosis, and AD, suggesting that the deleterious effects of TNFα mediated by the astrocytes could be common determinants of neurodegenerative processes (Liddelow et al., 2017).

## 7 DISCUSSION

### 7.1 The gliotransmission debate: New perspectives from single‐cell transcriptomic studies

The data presented in the previous Chapters strongly support the occurrence of glutamatergic gliotransmission at PP‐GC synapses in the DG. Evidence for gliotransmission at large, its implication in the rapid control of synaptic functions and plasticity, network oscillations and behavior (Santello et al., 2019) has been presented in many other circuits, in several brain regions, and shown to involve a variety of effects, transmitters and mechanisms (reviewed in Araque et al., 2014 and, more recently, in Noriega‐Prieto & Araque, 2021). Despite this extensive literature, the existence of gliotransmission has been repeatedly questioned over the last 15 years based on several types of criticism including: (a) Missing or unconvincing evidence for it; (b) existence of opposite “negative” evidence; (c) conceptual skepticism (e.g., Barres, 2008; Bazargani & Attwell, 2016; Fiacco et al., 2009; Nedergaard & Verkhratsky, 2012). As part of the ongoing debate, a summary of the two opposing viewpoints was presented in a “dual perspective” pair of reviews few years ago (Fiacco & McCarthy, 2018 vs Savtchouk & Volterra, 2018). Here we want to discuss more recent data that bring new information and perspective to the debate, particularly to the issue of glutamate exocytosis from astrocytes, pertinent to this review. In this context, one of the “negative” arguments presented in Fiacco & McCarthy, 2018 was the lack of transcriptomic and proteomic data supporting the presence in astrocytes of the molecular machinery needed for exocytosis of glutamate and other gliotransmitters, that is, the missing expression of VGLUTs and SNARE mRNA/proteins (Cahoy et al., 2008; Chai et al., 2017; Zhang et al., 2014). However, when considering “negative” transcriptomic or proteomic data, one should also consider the experimental protocols that brought to those data; for example, for transcriptomics, the fact that mRNA was extracted from pools of entire forebrain/cortices collected from several mice, at early time points in life (P1‐30), and RNA was sequenced using the RNAseq technology available at the time. Enormous methodological progress has been made since then, in particular moving from bulk transcriptomics to single‐cell approaches, with the introduction of single‐cell RNAseq (scRNAseq) technologies and protocols now allowing for full‐length coverage of cDNAs from individual astrocytes with high sensitivity (Stark et al., 2019). This new wave of single‐cell studies is revealing a much more complex landscape than the one presented by the pioneer bulk transcriptomics work, notably the existence of a large molecular heterogeneity of the astrocytes, depending on their developmental history, anatomical location, proposed circuit role and other factors. For example, astrocytes of the cerebral cortex were found to be molecularly diverse according to their laminar organization involving three separate layers (Bayraktar et al., 2020). Moreover, analysis of thousands of individual cortical and hippocampal astrocytes, led to their tentative classification in five molecularly distinct subtypes with proposed distinct biological roles (Batiuk et al., 2020). Importantly, these methodological advances also brought new numerical and conceptual information relevant to the glutamate gliotransmission debate. To start, even studies still performing bulk RNAseq analysis, but now from individual brain regions and at various ages ranging from the developmental period to the aged brain, for the first time reported detectable levels of VGLUTs and SNAREs (SNAP25, VAMP2 etc.) transcripts in astrocytes. Importantly, they showed that the levels ranged from below to above detection, with significant differences depending on region and age (e.g., Boisvert et al., 2018, see on‐line database http://igc1.salk.edu:3838/astrocyte_aging_transcriptome/). scRNAseq studies added the critical extra information that VGLUTs and SNARE transcripts are present in subpopulations of astrocytes and that these subpopulations have different regional and even sub‐regional distributions (e.g., Saunders et al., 2018, see on‐line database http://dropviz.org/). These data allow for new interpretations of the negative results. Thus, astrocytes expressing VGLUTs or SNARE transcripts appear to be a subgroup of the total astrocytic population and may have been missed by the initial bulk transcriptomics studies. Indeed, those studies collected and analyzed astrocytes as a single bulk population from a large portion of the brain at a given age, thereby most likely diluting out the subtle differences in expression of subsets of transcripts that characterize the local heterogeneity of astrocytes and its dynamics now revealed by single cell studies. In synthesis, initial bulk transcriptomic studies provided an average view of the astrocyte population, not a detailed one. Concerning HDML astrocytes expressing VGLUTs and SNAREs, we presented several lines of evidence congruent with the current single‐cell transcriptomic data. Thus, in Bezzi et al., 2004 we stated that: (1) 25% of the sampled astrocytes (P35‐70) presented VGLUTs transcripts in single cell RT‐PCR experiments; (2) 35% of the astrocytic processes (in adult HDML) analyzed by immunogold EM presented immunoreactivity for either VGLUT1 or VGLUT2 and for VAMP3; moreover, in Jourdain et al., 2007 we stated that: (3) in 1/3 of whole‐cell patched pairs of an HDML astrocyte and a GC, stimulation of the astrocyte induced synaptic potentiation in the GC, abolished by perfusion of the vesicular exocytosis blocker TeNT_LC_ in the astrocyte. Therefore, most likely only part of the astrocytes in the HDML release glutamate and exert synaptic regulatory effects. This conclusion fits with the emerging molecular heterogeneity of the astrocytes and with the view that gliotransmission may serve circuit‐specific regulatory functions, as seen not only in DG but also in other brain regions (e.g., Martín et al., 2015). Functional specificity seems to match emerging molecular specificity, for example, the presence of molecularly specialized “glutamatergic” or “GABAergic” astrocyte subtypes (Batiuk et al., 2020). The above study did not only identify the existence of these two specialized astrocytic subtypes, but also provided an initial in situ hybridization map of their anatomical locations opening to new studies aiming at addressing the reasons of their specificity. Certainly, much more information in this direction will come soon, brought by the rapid ongoing development of sensitive and precise spatial transcriptomic approaches (Longo et al., 2021).

### 7.2 Presynaptic NMDARs and functional implications: Divergent control of MEC‐DG and LEC‐DG circuits by astrocytes

A key determinant of the specific control exerted by astrocytes on the MEC‐DG circuit is the selective presence of “specialized” preNMDA receptors at MPP but not LPP terminals (Savtchouk et al., 2019). In addition to playing their ubiquitous role in the control of synaptic transmission and plasticity at postsynaptic sites, NMDARs are expressed also presynaptically in several brain regions, often in a synapse‐specific (Bouvier et al., 2015) and developmentally‐regulated manner (Hansen et al., 2018; Pérez‐Otaño et al., 2016). For example, in the somatosensory cortex of juvenile rats, NR2B‐containing preNMDARs are present at L4–L2/3 excitatory synapses, but not at other synapses made by L4 cells (L4–L4 synapses) or at other inputs onto L2/3 cells (synapses of the cross‐columnar L2/3–L2/3 projection) (Brasier & Feldman, 2008). In this case, the circuit‐specific expression of preNMDARs would promote preferential ascending activation of single S1 columns, relative to lateral spread of excitation across columns. In our case, activation of GluN3A‐containing pre‐NMDARs at MPP terminals could change the gain of presynaptic release relative to LPP axons and thereby contribute to reshape dendritic filtering and synaptic integration in GCs. This could be required, for instance, because of the different electrotonic distance of MPP and LPP synaptic inputs from the GC soma and/or their different spike timing onset, dictated by the intrinsically different activity patterns of the originating cells. It could also reflect the need to process MPP and LPP inputs onto GCs in a different manner and help to separate the different nature of the information they convey (Hainmueller & Bartos, 2020, see also Chapter 1). Intriguingly, a recent study reported that adult‐born GCs exert a modulatory action on mature GC excitability that has opposite effect at MPP‐GC versus LPP‐GC synapses. This modulatory action could serve to rapidly shift the balance between LPP‐mediated contextual information and MPP‐mediated spatial information processed by the GCs (Luna et al., 2019). Thus, during a novel object recognition task, accompanied by preferential firing of LPP axons, adult‐born GCs were driven by LPP to reduce excitation of mature GCs via activation of inhibitory group II mGluRs. In contrast, during an active place avoidance task, preferential firing from MPP axons drove adult‐born GCs to increase excitation of mature GCs via glutamatergic activation of GluN3‐containing NMDARs. Noteworthy, this latter excitatory control on MPP‐GC synapses and its GluN3‐dependent mechanism strikingly resemble those described by us, except for their attribution to an action of adult‐born GCs rather than astrocytes. Luna et al observations and ours' observations seem inconsistent in this key aspect, but this may not be the case. Indeed, Luna et al. conclude that adult‐born GCs directly connect with mature GCs because their modulatory effect is TTX‐insensitive, but this data per se does not exclude that the connection is instead made via an intermediary astrocyte (and astrocytes are known to promote adult‐born GCs connectivity, Krzisch et al., 2015).

### 7.3 HDML astrocytes and MPP terminals form direct connections?

Several data suggest that astrocytes and MPP terminals form direct connections, functionally and possibly also anatomically. Among the supporting evidence: (1) preNMDAR subunits at MPP terminals, both GluN2B (Jourdain et al., 2007) and GluN3A (Savtchouk et al., 2019), are concentrated at extra‐synaptic sites directly facing astrocytes and away from the synaptic cleft. (2) In keeping, preNMDARs do not respond to miniature synaptic release events, that is, are not easily accessed by synaptic glutamate (Savtchouk et al., 2019). (3) The MPP terminal membrane expressing preNMDARs and the facing astrocyte membrane are often separated by synaptic‐like distance, and the astrocyte membrane contains vesicular organelles (Bezzi et al., 2004; Jourdain et al., 2007). (4) The astrocytic membrane additionally expresses regularly spaced glutamate uptake molecules (Bezzi et al., 2004), which likely control glutamate levels at preNMDARs, allowing for their activation from local sources while shielding them from more distal ones. (5) PreNMDARs at MPP terminals express the GluN3A subunit, which makes them poorly Mg^2+^ sensitive and amenable to activation by astrocytes independent of coincident firing of MPP axons.

The high level of structural and functional organization seen at the extrasynaptic sites of MPP terminals and their facing astrocytic membranes, suggests that astrocytes and synaptic elements could possess sites of direct connectivity and communication, like neuron–neuron synapses (Araque et al., 2014). Recently this view has been reinforced by observing the molecular organization put in place at strategic places of astrocyte‐neuron contact where dyads of adhesion factors (e.g., ephrins/Eph receptors; neuroligins/neurexins), one expressed on the astrocyte membrane and the other on the neuronal membrane, bind each other and physically connect the two membranes (reviewed in Tan & Eroglu, 2021). Even more stringently, combined ultrastructural and functional evidence indicates the existence of authentic synapses between neurons and glia (e.g., NG2 cells), in all like classic neuronal synapses (reviewed in Bergles et al., 2010). In this context, an intriguing observation directly relevant to the astrocyte case, was made in a retrograde labelling study using rabies virus (RABV)‐based tracing. The study reported that RABV, present in EC neurons, labeled a sub‐fraction of HDML astrocytes (Schwarz et al., 2015). Since, in general, RABV jumps in a mono‐synaptic retrograde fashion from a postsynaptic neuron to its presynaptic counterpart, these data would imply that astrocytes are presynaptic to PP fibers of EC neurons, in line with our functional data and with the idea that astrocytes are directly connected in “synaptic‐like” way to MPP terminals.

Overall, the above data outline the possible existence of astrocyte‐neuron synaptic‐like connections at PP‐GC synapses, an idea that conceptually challenges our current understanding of astrocytes and their roles in synaptic circuits. Further rigorous ad hoc investigations are, however, required to provide a solid demonstration of this atypical connectivity and its function as well as to answer the additional pending aspects discussed in this review about astrocytes in EC‐DG memory circuit function.

## CONFLICT OF INTEREST

The authors declare no conflicts of interest.

## AUTHOR CONTRIBUTION

Maria Amalia Di Castro and Andrea Volterra wrote this review manuscript.

## Data Availability

Data sharing is not applicable to this article as no new data were created or analyzed in this study.

## References

[glia24128-bib-0001] Agarwal, A. , Wu, P. H. , Hughes, E. G. , Fukaya, M. , Tischfield, M. A. , Langseth, A. J. , Wirtz, D. , & Bergles, D. E. (2017). Transient opening of the mitochondrial permeability transition pore induces microdomain calcium transients in astrocyte processes. Neuron, 93(3), 587–605.e7. 10.1016/j.neuron.2016.12.034 28132831 PMC5308886

[glia24128-bib-0002] Akers, K. G. , Martinez‐Canabal, A. , Restivo, L. , Yiu, A. P. , De Cristofaro, A. , Hsiang, H. L. , Wheeler, A. L. , Guskjolen, A. , Niibori, Y. , Shoji, H. , Ohira, K. , Richards, B. A. , Miyakawa, T. , Josselyn, S. A. , & Frankland, P. W. (2014). Hippocampal neurogenesis regulates forgetting during adulthood and infancy. Science (New York, N.Y.), 344(6184), 598–602. 10.1126/science.1248903 24812394

[glia24128-bib-0003] Araque, A. , Carmignoto, G. , Haydon, P. G. , Oliet, S. H. , Robitaille, R. , & Volterra, A. (2014). Gliotransmitters travel in time and space. Neuron, 81(4), 728–739. 10.1016/j.neuron.2014.02.007 24559669 PMC4107238

[glia24128-bib-0004] Araque, A. , Parpura, V. , Sanzgiri, R. P. , & Haydon, P. G. (1999). Tripartite synapses: Glia, the unacknowledged partner. Trends in Neurosciences, 22(5), 208–215. 10.1016/s0166-2236(98)01349-6 10322493

[glia24128-bib-0005] Arizono, M. , Inavalli, V. , Panatier, A. , Pfeiffer, T. , Angibaud, J. , Levet, F. , Ter Veer, M. , Stobart, J. , Bellocchio, L. , Mikoshiba, K. , Marsicano, G. , Weber, B. , Oliet, S. , & Nägerl, U. V. (2020). Structural basis of astrocytic Ca^2+^ signals at tripartite synapses. Nature Communications, 11(1), 1906. 10.1038/s41467-020-15648-4 PMC7170846 32312988

[glia24128-bib-0006] Barres, B. A. (2008). The mystery and magic of glia: A perspective on their roles in health and disease. Neuron, 60(3), 430–440. 10.1016/j.neuron.2008.10.013 18995817

[glia24128-bib-0007] Batiuk, M. Y. , Martirosyan, A. , Wahis, J. , de Vin, F. , Marneffe, C. , Kusserow, C. , Koeppen, J. , Viana, J. F. , Oliveira, J. F. , Voet, T. , Ponting, C. P. , Belgard, T. G. , & Holt, M. G. (2020). Identification of region‐specific astrocyte subtypes at single cell resolution. Nature Communications, 11(1), 1220. 10.1038/s41467-019-14198-8 PMC7058027 32139688

[glia24128-bib-0008] Bayraktar, O. A. , Bartels, T. , Holmqvist, S. , Kleshchevnikov, V. , Martirosyan, A. , Polioudakis, D. , Ben Haim, L. , Young, A. , Batiuk, M. Y. , Prakash, K. , Brown, A. , Roberts, K. , Paredes, M. F. , Kawaguchi, R. , Stockley, J. H. , Sabeur, K. , Chang, S. M. , Huang, E. , Hutchinson, P. , … Rowitch, D. H. (2020). Astrocyte layers in the mammalian cerebral cortex revealed by a single‐cell in situ transcriptomic map. Nature Neuroscience, 23(4), 500–509. 10.1038/s41593-020-0602-1 32203496 PMC7116562

[glia24128-bib-0009] Bazargani, N. , & Attwell, D. (2016). Astrocyte calcium signaling: The third wave. Nature Neuroscience, 19(2), 182–189. 10.1038/nn.4201 26814587

[glia24128-bib-0010] Beattie, E. C. , Stellwagen, D. , Morishita, W. , Bresnahan, J. C. , Ha, B. K. , Von Zastrow, M. , Beattie, M. S. , & Malenka, R. C. (2002). Control of synaptic strength by glial TNFalpha. Science (New York, N.Y.), 295(5563), 2282–2285. 10.1126/science.1067859 11910117

[glia24128-bib-0011] Bergersen, L. H. , Morland, C. , Ormel, L. , Rinholm, J. E. , Larsson, M. , Wold, J. F. , Røe, A. T. , Stranna, A. , Santello, M. , Bouvier, D. , Ottersen, O. P. , Volterra, A. , & Gundersen, V. (2012). Immunogold detection of L‐glutamate and D‐serine in small synaptic‐like microvesicles in adult hippocampal astrocytes. Cerebral cortex (New York, N.Y.: 1991), 22(7), 1690–1697. 10.1093/cercor/bhr254 21914633

[glia24128-bib-0012] Bergles, D. E. , Jabs, R. , & Steinhäuser, C. (2010). Neuron‐glia synapses in the brain. Brain Research Reviews, 63(1–2), 130–137. 10.1016/j.brainresrev.2009.12.003 20018210 PMC2862892

[glia24128-bib-0013] Bernardinelli, Y. , Randall, J. , Janett, E. , Nikonenko, I. , König, S. , Jones, E. V. , Flores, C. E. , Murai, K. K. , Bochet, C. G. , Holtmaat, A. , & Muller, D. (2014). Activity‐dependent structural plasticity of perisynaptic astrocytic domains promotes excitatory synapse stability. Current biology: CB, 24(15), 1679–1688. 10.1016/j.cub.2014.06.025 25042585

[glia24128-bib-0014] Bezzi, P. , Carmignoto, G. , Pasti, L. , Vesce, S. , Rossi, D. , Rizzini, B. L. , Pozzan, T. , & Volterra, A. (1998). Prostaglandins stimulate calcium‐dependent glutamate release in astrocytes. Nature, 391(6664), 281–285. 10.1038/34651 9440691

[glia24128-bib-0015] Bezzi, P. , Domercq, M. , Brambilla, L. , Galli, R. , Schols, D. , De Clercq, E. , Vescovi, A. , Bagetta, G. , Kollias, G. , Meldolesi, J. , & Volterra, A. (2001). CXCR4‐activated astrocyte glutamate release via TNFalpha: Amplification by microglia triggers neurotoxicity. Nature Neuroscience, 4(7), 702–710. 10.1038/89490 11426226

[glia24128-bib-0016] Bezzi, P. , Gundersen, V. , Galbete, J. L. , Seifert, G. , Steinhäuser, C. , Pilati, E. , & Volterra, A. (2004). Astrocytes contain a vesicular compartment that is competent for regulated exocytosis of glutamate. Nature Neuroscience, 7(6), 613–620. 10.1038/nn1246 15156145

[glia24128-bib-0017] Bindocci, E. , Savtchouk, I. , Liaudet, N. , Becker, D. , Carriero, G. , & Volterra, A. (2017). Three‐dimensional Ca^2+^ imaging advances understanding of astrocyte biology. Science (New York, N.Y.), 356(6339), eaai8185. 10.1126/science.aai8185 28522470

[glia24128-bib-0018] Boisvert, M. M. , Erikson, G. A. , Shokhirev, M. N. , & Allen, N. J. (2018). The aging astrocyte Transcriptome from multiple regions of the mouse brain. Cell Reports, 22(1), 269–285. 10.1016/j.celrep.2017.12.039 29298427 PMC5783200

[glia24128-bib-0019] Boldrini, M. , Fulmore, C. A. , Tartt, A. N. , Simeon, L. R. , Pavlova, I. , Poposka, V. , Rosoklija, G. B. , Stankov, A. , Arango, V. , Dwork, A. J. , Hen, R. , & Mann, J. J. (2018). Human hippocampal neurogenesis persists throughout aging. Cell Stem Cell, 22(4), 589–599.e5. 10.1016/j.stem.2018.03.015 29625071 PMC5957089

[glia24128-bib-0020] Bouvier, G. , Bidoret, C. , Casado, M. , & Paoletti, P. (2015). Presynaptic NMDA receptors: Roles and rules. Neuroscience, 311, 322–340. 10.1016/j.neuroscience.2015.10.033 26597763

[glia24128-bib-0021] Bowser, D. N. , & Khakh, B. S. (2007). Two forms of single‐vesicle astrocyte exocytosis imaged with total internal reflection fluorescence microscopy. Proceedings of the National Academy of Sciences of the United States of America, 104(10), 4212–4217. 10.1073/pnas.0607625104 17360502 PMC1820734

[glia24128-bib-0022] Brasier, D. J. , & Feldman, D. E. (2008). Synapse‐specific expression of functional presynaptic NMDA receptors in rat somatosensory cortex. The Journal of Neuroscience: The Official Journal of the Society for Neuroscience, 28(9), 2199–2211. 10.1523/JNEUROSCI.3915-07.2008 18305253 PMC3071744

[glia24128-bib-0023] Burwell, R. D. , Saddoris, M. P. , Bucci, D. J. , & Wiig, K. A. (2004). Corticohippocampal contributions to spatial and contextual learning. The Journal of Neuroscience: The Official Journal of the Society for Neuroscience, 24(15), 3826–3836. 10.1523/JNEUROSCI.0410-04.2004 15084664 PMC6729354

[glia24128-bib-0024] Cahoy, J. D. , Emery, B. , Kaushal, A. , Foo, L. C. , Zamanian, J. L. , Christopherson, K. S. , Xing, Y. , Lubischer, J. L. , Krieg, P. A. , Krupenko, S. A. , Thompson, W. J. , & Barres, B. A. (2008). A transcriptome database for astrocytes, neurons, and oligodendrocytes: A new resource for understanding brain development and function. The Journal of neuroscience: The official journal of the Society for Neuroscience, 28(1), 264–278. 10.1523/JNEUROSCI.4178-07.2008 18171944 PMC6671143

[glia24128-bib-0025] Chai, H. , Diaz‐Castro, B. , Shigetomi, E. , Monte, E. , Octeau, J. C. , Yu, X. , Cohn, W. , Rajendran, P. S. , Vondriska, T. M. , Whitelegge, J. P. , Coppola, G. , & Khakh, B. S. (2017). Neural circuit‐specialized astrocytes: Transcriptomic, proteomic, morphological, and functional evidence. Neuron, 95(3), 531–549.e9. 10.1016/j.neuron.2017.06.029 28712653 PMC5811312

[glia24128-bib-0026] Chiaravalloti, N. D. , & DeLuca, J. (2008). Cognitive impairment in multiple sclerosis. The Lancet. Neurology, 7(12), 1139–1151. 10.1016/S1474-4422(08)70259-X 19007738

[glia24128-bib-0027] Christie, B. R. , & Abraham, W. C. (1992a). Priming of associative long‐term depression in the dentate gyrus by theta frequency synaptic activity. Neuron, 9(1), 79–84. 10.1016/0896-6273(92)90222-y 1321647

[glia24128-bib-0028] Christie, B. R. , & Abraham, W. C. (1992b). NMDA‐dependent heterosynaptic long‐term depression in the dentate gyrus of anaesthetized rats. Synapse (New York, N.Y.), 10(1), 1–6. 10.1002/syn.890100102 1531559

[glia24128-bib-0029] Clark, I. A. , Alleva, L. M. , & Vissel, B. (2010). The roles of TNF in brain dysfunction and disease. Pharmacology & Therapeutics, 128(3), 519–548. 10.1016/j.pharmthera.2010.08.007 20813131

[glia24128-bib-0030] Clelland, C. D. , Choi, M. , Romberg, C. , Clemenson, G. D., Jr. , Fragniere, A. , Tyers, P. , Jessberger, S. , Saksida, L. M. , Barker, R. A. , Gage, F. H. , & Bussey, T. J. (2009). A functional role for adult hippocampal neurogenesis in spatial pattern separation. Science (New York, N.Y.), 325(5937), 210–213. 10.1126/science.1173215 PMC2997634 19590004

[glia24128-bib-0031] Colino, A. , & Malenka, R. C. (1993). Mechanisms underlying induction of long‐term potentiation in rat medial and lateral perforant paths in vitro. Journal of Neurophysiology, 69(4), 1150–1159. 10.1152/jn.1993.69.4.1150 8492154

[glia24128-bib-0032] Delekate, A. , Füchtemeier, M. , Schumacher, T. , Ulbrich, C. , Foddis, M. , & Petzold, G. C. (2014). Metabotropic P2Y1 receptor signalling mediates astrocytic hyperactivity in vivo in an Alzheimer's disease mouse model. Nature Communications, 5, 5422. 10.1038/ncomms6422 25406732

[glia24128-bib-0033] Deng, W. , Aimone, J. B. , & Gage, F. H. (2010). New neurons and new memories: How does adult hippocampal neurogenesis affect learning and memory? Nature Reviews. Neuroscience, 11(5), 339–350. 10.1038/nrn2822 20354534 PMC2886712

[glia24128-bib-0034] Deng, W. , Saxe, M. D. , Gallina, I. S. , & Gage, F. H. (2009). Adult‐born hippocampal dentate granule cells undergoing maturation modulate learning and memory in the brain. The Journal of Neuroscience: The Official Journal of the Society for Neuroscience, 29(43), 13532–13542. 10.1523/JNEUROSCI.3362-09.2009 19864566 PMC2787190

[glia24128-bib-0035] Di Castro, M. A. , Chuquet, J. , Liaudet, N. , Bhaukaurally, K. , Santello, M. , Bouvier, D. , Tiret, P. , & Volterra, A. (2011). Local Ca^2+^ detection and modulation of synaptic release by astrocytes. Nature Neuroscience, 14(10), 1276–1284. 10.1038/nn.2929 21909085

[glia24128-bib-0036] Dolorfo, C. L. , & Amaral, D. G. (1998a). Entorhinal cortex of the rat: Organization of intrinsic connections. The Journal of Comparative Neurology, 398(1), 49–82. 10.1002/(sici)1096-9861(19980817)398:1<49::aid-cne4>3.0.co;2-9 9703027

[glia24128-bib-0037] Dolorfo, C. L. , & Amaral, D. G. (1998b). Entorhinal cortex of the rat: Topographic organization of the cells of origin of the perforant path projection to the dentate gyrus. The Journal of Comparative Neurology, 398(1), 25–48. 9703026

[glia24128-bib-0038] Domercq, M. , Brambilla, L. , Pilati, E. , Marchaland, J. , Volterra, A. , & Bezzi, P. (2006). P2Y1 receptor‐evoked glutamate exocytosis from astrocytes: Control by tumor necrosis factor‐alpha and prostaglandins. The Journal of Biological Chemistry, 281(41), 30684–30696. 10.1074/jbc.M606429200 16882655

[glia24128-bib-0040] Eriksson, P. S. , Perfilieva, E. , Björk‐Eriksson, T. , Alborn, A. M. , Nordborg, C. , Peterson, D. A. , & Gage, F. H. (1998). Neurogenesis in the adult human hippocampus. Nature Medicine, 4(11), 1313–1317. 10.1038/3305 9809557

[glia24128-bib-0041] Ferbinteanu, J. , Holsinger, R. M. , & McDonald, R. J. (1999). Lesions of the medial or lateral perforant path have different effects on hippocampal contributions to place learning and on fear conditioning to context. Behavioural Brain Research, 101(1), 65–84. 10.1016/s0166-4328(98)00144-2 10342401

[glia24128-bib-0042] Fiacco, T. A. , Agulhon, C. , & McCarthy, K. D. (2009). Sorting out astrocyte physiology from pharmacology. Annual Review of Pharmacology and Toxicology, 49, 151–174. 10.1146/annurev.pharmtox.011008.145602 18834310

[glia24128-bib-0043] Fiacco, T. A. , & McCarthy, K. D. (2018). Multiple Lines of evidence indicate that Gliotransmission does not occur under physiological conditions. The Journal of neuroscience: The official journal of the Society for Neuroscience, 38(1), 3–13. 10.1523/JNEUROSCI.0016-17.2017 29298904 PMC5761435

[glia24128-bib-0044] Froc, D. J. , Eadie, B. , Li, A. M. , Wodtke, K. , Tse, M. , & Christie, B. R. (2003). Reduced synaptic plasticity in the lateral perforant path input to the dentate gyrus of aged C57BL/6 mice. Journal of Neurophysiology, 90(1), 32–38. 10.1152/jn.00105.2003 12634277

[glia24128-bib-0045] Gómez‐Gonzalo, M. , Martin‐Fernandez, M. , Martínez‐Murillo, R. , Mederos, S. , Hernández‐Vivanco, A. , Jamison, S. , Fernandez, A. P. , Serrano, J. , Calero, P. , Futch, H. S. , Corpas, R. , Sanfeliu, C. , Perea, G. , & Araque, A. (2017). Neuron‐astrocyte signaling is preserved in the aging brain. Glia, 65(4), 569–580. 10.1002/glia.23112 28130845 PMC5314210

[glia24128-bib-0046] Gómez‐Isla, T. , Price, J. L. , McKeel, D. W., Jr. , Morris, J. C. , Growdon, J. H. , & Hyman, B. T. (1996). Profound loss of layer II entorhinal cortex neurons occurs in very mild Alzheimer's disease. The Journal of Neuroscience: The Official Journal of the Society for Neuroscience, 16(14), 4491–4500. 10.1523/JNEUROSCI.16-14-04491.1996 8699259 PMC6578866

[glia24128-bib-0047] Götz, J. , Bodea, L. G. , & Goedert, M. (2018). Rodent models for Alzheimer disease. Nature Reviews. Neuroscience, 19(10), 583–598. 10.1038/s41583-018-0054-8 30194347

[glia24128-bib-0048] Gould, E. , Beylin, A. , Tanapat, P. , Reeves, A. , & Shors, T. J. (1999). Learning enhances adult neurogenesis in the hippocampal formation. Nature Neuroscience, 2(3), 260–265. 10.1038/6365 10195219

[glia24128-bib-0049] Habbas, S. , Santello, M. , Becker, D. , Stubbe, H. , Zappia, G. , Liaudet, N. , Klaus, F. R. , Kollias, G. , Fontana, A. , Pryce, C. R. , Suter, T. , & Volterra, A. (2015). Neuroinflammatory TNFα impairs memory via astrocyte signaling. Cell, 163(7), 1730–1741. 10.1016/j.cell.2015.11.023 26686654

[glia24128-bib-0050] Hafting, T. , Fyhn, M. , Molden, S. , Moser, M. B. , & Moser, E. I. (2005). Microstructure of a spatial map in the entorhinal cortex. Nature, 436(7052), 801–806. 10.1038/nature03721 15965463

[glia24128-bib-0051] Hainmueller, T. , & Bartos, M. (2020). Dentate gyrus circuits for encoding, retrieval and discrimination of episodic memories. Nature Reviews. Neuroscience, 21(3), 153–168. 10.1038/s41583-019-0260-z 32042144 PMC7115869

[glia24128-bib-0052] Hamilton, N. B. , & Attwell, D. (2010). Do astrocytes really exocytose neurotransmitters? Nature Reviews. Neuroscience, 11(4), 227–238. 10.1038/nrn2803 20300101

[glia24128-bib-0053] Hansen, K. B. , Yi, F. , Perszyk, R. E. , Furukawa, H. , Wollmuth, L. P. , Gibb, A. J. , & Traynelis, S. F. (2018). Structure, function, and allosteric modulation of NMDA receptors. The Journal of General Physiology, 150(8), 1081–1105. 10.1085/jgp.201812032 30037851 PMC6080888

[glia24128-bib-0054] Hargreaves, E. L. , Rao, G. , Lee, I. , & Knierim, J. J. (2005). Major dissociation between medial and lateral entorhinal input to dorsal hippocampus. Science (New York, N.Y.), 308(5729), 1792–1794. 10.1126/science.1110449 15961670

[glia24128-bib-0055] Haustein, M. D. , Kracun, S. , Lu, X. H. , Shih, T. , Jackson‐Weaver, O. , Tong, X. , Xu, J. , Yang, X. W. , O'Dell, T. J. , Marvin, J. S. , Ellisman, M. H. , Bushong, E. A. , Looger, L. L. , & Khakh, B. S. (2014). Conditions and constraints for astrocyte calcium signaling in the hippocampal mossy fiber pathway. Neuron, 82(2), 413–429. 10.1016/j.neuron.2014.02.041 24742463 PMC4086217

[glia24128-bib-0056] Haydon, P. G. (2001). GLIA: Listening and talking to the synapse. Nature Reviews. Neuroscience, 2(3), 185–193. 10.1038/35058528 11256079

[glia24128-bib-0057] He, P. , Zhong, Z. , Lindholm, K. , Berning, L. , Lee, W. , Lemere, C. , Staufenbiel, M. , Li, R. , & Shen, Y. (2007). Deletion of tumor necrosis factor death receptor inhibits amyloid beta generation and prevents learning and memory deficits in Alzheimer's mice. The Journal of Cell Biology, 178(5), 829–841. 10.1083/jcb.200705042 17724122 PMC2064547

[glia24128-bib-0058] Henson, M. A. , Roberts, A. C. , Pérez‐Otaño, I. , & Philpot, B. D. (2010). Influence of the NR3A subunit on NMDA receptor functions. Progress in Neurobiology, 91(1), 23–37. 10.1016/j.pneurobio.2010.01.004 20097255 PMC2883719

[glia24128-bib-0059] Hunsaker, M. R. , Mooy, G. G. , Swift, J. S. , & Kesner, R. P. (2007). Dissociations of the medial and lateral perforant path projections into dorsal DG, CA3, and CA1 for spatial and nonspatial (visual object) information processing. Behavioral Neuroscience, 121(4), 742–750. 10.1037/0735-7044.121.4.742 17663599

[glia24128-bib-0060] Hyman, B. T. , Van Hoesen, G. W. , Damasio, A. R. , & Barnes, C. L. (1984). Alzheimer's disease: Cell‐specific pathology isolates the hippocampal formation. Science (New York, N.Y.), 225(4667), 1168–1170. 10.1126/science.6474172 6474172

[glia24128-bib-0061] Jacobsen, J. S. , Wu, C. C. , Redwine, J. M. , Comery, T. A. , Arias, R. , Bowlby, M. , Martone, R. , Morrison, J. H. , Pangalos, M. N. , Reinhart, P. H. , & Bloom, F. E. (2006). Early‐onset behavioral and synaptic deficits in a mouse model of Alzheimer's disease. Proceedings of the National Academy of Sciences of the United States of America, 103(13), 5161–5166. 10.1073/pnas.0600948103 16549764 PMC1405622

[glia24128-bib-0062] Jiang, N. , Cupolillo, D. , Grosjean, N. , Muller, E. , Deforges, S. , Mulle, C. , & Amédée, T. (2021). Impaired plasticity of intrinsic excitability in the dentate gyrus alters spike transfer in a mouse model of Alzheimer's disease. Neurobiology of Disease, 154, 105345. 10.1016/j.nbd.2021.105345 33766653

[glia24128-bib-0063] Jourdain, P. , Bergersen, L. H. , Bhaukaurally, K. , Bezzi, P. , Santello, M. , Domercq, M. , Matute, C. , Tonello, F. , Gundersen, V. , & Volterra, A. (2007). Glutamate exocytosis from astrocytes controls synaptic strength. Nature Neuroscience, 10(3), 331–339. 10.1038/nn1849 17310248

[glia24128-bib-0064] Kaeser, P. S. , & Regehr, W. G. (2017). The readily releasable pool of synaptic vesicles. Current Opinion in Neurobiology, 43, 63–70. 10.1016/j.conb.2016.12.012 28103533 PMC5447466

[glia24128-bib-0065] Kahle, J. S. , & Cotman, C. W. (1989). Carbachol depresses synaptic responses in the medial but not the lateral perforant path. Brain Research, 482(1), 159–163. 10.1016/0006-8993(89)90554-4 2706473

[glia24128-bib-0066] Kempermann, G. , Kuhn, H. G. , & Gage, F. H. (1997). More hippocampal neurons in adult mice living in an enriched environment. Nature, 386(6624), 493–495. 10.1038/386493a0 9087407

[glia24128-bib-0067] Khan, U. A. , Liu, L. , Provenzano, F. A. , Berman, D. E. , Profaci, C. P. , Sloan, R. , Mayeux, R. , Duff, K. E. , & Small, S. A. (2014). Molecular drivers and cortical spread of lateral entorhinal cortex dysfunction in preclinical Alzheimer's disease. Nature Neuroscience, 17(2), 304–311. 10.1038/nn.3606 24362760 PMC4044925

[glia24128-bib-0068] Knierim, J. J. , Lee, I. , & Hargreaves, E. L. (2006). Hippocampal place cells: Parallel input streams, subregional processing, and implications for episodic memory. Hippocampus, 16(9), 755–764. 10.1002/hipo.20203 16883558

[glia24128-bib-0069] Kofuji, P. , & Araque, A. (2021). G‐protein‐coupled receptors in astrocyte‐neuron communication. Neuroscience, 456, 71–84. 10.1016/j.neuroscience.2020.03.025 32224231 PMC8817509

[glia24128-bib-0070] Kohonen, T. (1984). Self‐organization and associative memory. Springer‐Verlag.

[glia24128-bib-0071] Krzisch, M. , Temprana, S. G. , Mongiat, L. A. , Armida, J. , Schmutz, V. , Virtanen, M. A. , Kocher‐Braissant, J. , Kraftsik, R. , Vutskits, L. , Conzelmann, K. K. , Bergami, M. , Gage, F. H. , Schinder, A. F. , & Toni, N. (2015). Pre‐existing astrocytes form functional perisynaptic processes on neurons generated in the adult hippocampus. Brain Structure & Function, 220(4), 2027–2042. 10.1007/s00429-014-0768-y 24748560 PMC4481333

[glia24128-bib-0072] Kuchibhotla, K. V. , Lattarulo, C. R. , Hyman, B. T. , & Bacskai, B. J. (2009). Synchronous hyperactivity and intercellular calcium waves in astrocytes in Alzheimer mice. Science (New York, N.Y.), 323(5918), 1211–1215. 10.1126/science.1169096 PMC2884172 19251629

[glia24128-bib-0073] Larsson, M. , Sawada, K. , Morland, C. , Hiasa, M. , Ormel, L. , Moriyama, Y. , & Gundersen, V. (2012). Functional and anatomical identification of a vesicular transporter mediating neuronal ATP release. Cerebral cortex (New York, N.Y.: 1991), 22(5), 1203–1214. 10.1093/cercor/bhr203 21810784

[glia24128-bib-0074] Liddelow, S. A. , Guttenplan, K. A. , Clarke, L. E. , Bennett, F. C. , Bohlen, C. J. , Schirmer, L. , Bennett, M. L. , Münch, A. E. , Chung, W. S. , Peterson, T. C. , Wilton, D. K. , Frouin, A. , Napier, B. A. , Panicker, N. , Kumar, M. , Buckwalter, M. S. , Rowitch, D. H. , Dawson, V. L. , Dawson, T. M. , … Barres, B. A. (2017). Neurotoxic reactive astrocytes are induced by activated microglia. Nature, 541(7638), 481–487. 10.1038/nature21029 28099414 PMC5404890

[glia24128-bib-0075] Lin, D. T. , Makino, Y. , Sharma, K. , Hayashi, T. , Neve, R. , Takamiya, K. , & Huganir, R. L. (2009). Regulation of AMPA receptor extrasynaptic insertion by 4.1N, phosphorylation and palmitoylation. Nature Neuroscience, 12(7), 879–887. 10.1038/nn.2351 19503082 PMC2712131

[glia24128-bib-0076] Liu, X. , Ramirez, S. , Pang, P. T. , Puryear, C. B. , Govindarajan, A. , Deisseroth, K. , & Tonegawa, S. (2012). Optogenetic stimulation of a hippocampal engram activates fear memory recall. Nature, 484(7394), 381–385. 10.1038/nature11028 22441246 PMC3331914

[glia24128-bib-0077] Longo, S. K. , Guo, M. G. , Ji, A. L. , & Khavari, P. A. (2021). Integrating single‐cell and spatial transcriptomics to elucidate intercellular tissue dynamics. Nature Reviews. Genetics, 22, 627–644. 10.1038/s41576-021-00370-8 PMC9888017 34145435

[glia24128-bib-0078] Luna, V. M. , Anacker, C. , Burghardt, N. S. , Khandaker, H. , Andreu, V. , Millette, A. , Leary, P. , Ravenelle, R. , Jimenez, J. C. , Mastrodonato, A. , Denny, C. A. , Fenton, A. A. , Scharfman, H. E. , & Hen, R. (2019). Adult‐born hippocampal neurons bidirectionally modulate entorhinal inputs into the dentate gyrus. Science (New York, N.Y.), 364(6440), 578–583. 10.1126/science.aat8789 PMC6800071 31073064

[glia24128-bib-0079] Macek, T. A. , Winder, D. G. , Gereau, R. W., 4th , Ladd, C. O. , & Conn, P. J. (1996). Differential involvement of group II and group III mGluRs as autoreceptors at lateral and medial perforant path synapses. Journal of Neurophysiology, 76(6), 3798–3806. 10.1152/jn.1996.76.6.3798 8985877

[glia24128-bib-0080] Marcantoni, A. , Raymond, E. F. , Carbone, E. , & Marie, H. (2014). Firing properties of entorhinal cortex neurons and early alterations in an Alzheimer's disease transgenic model. Pflugers Archiv: European journal of physiology, 466(7), 1437–1450. 10.1007/s00424-013-1368-z 24132829

[glia24128-bib-0081] Martín, R. , Bajo‐Grañeras, R. , Moratalla, R. , Perea, G. , & Araque, A. (2015). Circuit‐specific signaling in astrocyte‐neuron networks in basal ganglia pathways. Science (New York, N.Y.), 349(6249), 730–734. 10.1126/science.aaa7945 26273054

[glia24128-bib-0082] McKinney, R. A. , Capogna, M. , Dürr, R. , Gähwiler, B. H. , & Thompson, S. M. (1999). Miniature synaptic events maintain dendritic spines via AMPA receptor activation. Nature Neuroscience, 2(1), 44–49. 10.1038/4548 10195179

[glia24128-bib-0083] McNaughton, B. L. (1980). Evidence for two physiologically distinct perforant pathways to the fascia dentata. Brain Research, 199(1), 1–19. 10.1016/0006-8993(80)90226-7 7407615

[glia24128-bib-0084] McNaughton, B. L. , & Barnes, C. A. (1977). Physiological identification and analysis of dentate granule cell responses to stimulation of the medial and lateral perforant pathways in the rat. The Journal of Comparative Neurology, 175(4), 439–454. 10.1002/cne.901750404 915033

[glia24128-bib-0085] Min, M. Y. , Asztely, F. , Kokaia, M. , & Kullmann, D. M. (1998). Long‐term potentiation and dual‐component quantal signaling in the dentate gyrus. Proceedings of the National Academy of Sciences of the United States of America, 95(8), 4702–4707. 10.1073/pnas.95.8.4702 9539802 PMC22554

[glia24128-bib-0086] Mohamad, O. , Song, M. , Wei, L. , & Yu, S. P. (2013). Regulatory roles of the NMDA receptor GluN3A subunit in locomotion, pain perception and cognitive functions in adult mice. The Journal of Physiology, 591(1), 149–168. 10.1113/jphysiol.2012.239251 23006484 PMC3630778

[glia24128-bib-0087] Moreno‐Jiménez, E. P. , Flor‐García, M. , Terreros‐Roncal, J. , Rábano, A. , Cafini, F. , Pallas‐Bazarra, N. , Ávila, J. , & Llorens‐Martín, M. (2019). Adult hippocampal neurogenesis is abundant in neurologically healthy subjects and drops sharply in patients with Alzheimer's disease. Nature Medicine, 25(4), 554–560. 10.1038/s41591-019-0375-9 30911133

[glia24128-bib-0088] Moreno‐Jiménez, E. P. , Terreros‐Roncal, J. , Flor‐García, M. , Rábano, A. , & Llorens‐Martín, M. (2021). Evidences for adult hippocampal neurogenesis in humans. The Journal of Neuroscience: The Official Journal of the Society for Neuroscience, 41(12), 2541–2553. 10.1523/JNEUROSCI.0675-20.2020 33762406 PMC8018741

[glia24128-bib-0089] Moser, E. I. , Moser, M. B. , & McNaughton, B. L. (2017). Spatial representation in the hippocampal formation: A history. Nature Neuroscience, 20(11), 1448–1464. 10.1038/nn.4653 29073644

[glia24128-bib-0090] Nedergaard, M. , & Verkhratsky, A. (2012). Artifact versus reality–how astrocytes contribute to synaptic events. Glia, 60(7), 1013–1023. 10.1002/glia.22288 22228580 PMC3340515

[glia24128-bib-0091] Noriega‐Prieto, J. A. , & Araque, A. (2021). Sensing and regulating synaptic activity by astrocytes at tripartite synapse. Neurochemical Research, 46, 2580–2585. 10.1007/s11064-021-03317-x 33837868 PMC10159683

[glia24128-bib-0092] Padamsey, Z. , Tong, R. , & Emptage, N. (2017). Glutamate is required for depression but not potentiation of long‐term presynaptic function. eLife, 6, e29688. 10.7554/eLife.29688 29140248 PMC5714480

[glia24128-bib-0093] Palop, J. J. , Chin, J. , Roberson, E. D. , Wang, J. , Thwin, M. T. , Bien‐Ly, N. , Yoo, J. , Ho, K. O. , Yu, G. Q. , Kreitzer, A. , Finkbeiner, S. , Noebels, J. L. , & Mucke, L. (2007). Aberrant excitatory neuronal activity and compensatory remodeling of inhibitory hippocampal circuits in mouse models of Alzheimer's disease. Neuron, 55(5), 697–711. 10.1016/j.neuron.2007.07.025 17785178 PMC8055171

[glia24128-bib-0094] Panatier, A. , Vallée, J. , Haber, M. , Murai, K. K. , Lacaille, J. C. , & Robitaille, R. (2011). Astrocytes are endogenous regulators of basal transmission at central synapses. Cell, 146(5), 785–798. 10.1016/j.cell.2011.07.022 21855979

[glia24128-bib-0095] Pankratov, Y. , Lalo, U. , Verkhratsky, A. , & North, R. A. (2006). Vesicular release of ATP at central synapses. Pflugers Archiv: European Journal of Physiology, 452(5), 589–597. 10.1007/s00424-006-0061-x 16639550

[glia24128-bib-0096] Paoletti, P. , Bellone, C. , & Zhou, Q. (2013). NMDA receptor subunit diversity: Impact on receptor properties, synaptic plasticity and disease. Nature Reviews. Neuroscience, 14(6), 383–400. 10.1038/nrn3504 23686171

[glia24128-bib-0097] Pasti, L. , Volterra, A. , Pozzan, T. , & Carmignoto, G. (1997). Intracellular calcium oscillations in astrocytes: A highly plastic, bidirectional form of communication between neurons and astrocytes in situ. The Journal of Neuroscience: The Official Journal of the Society for Neuroscience, 17(20), 7817–7830. 10.1523/JNEUROSCI.17-20-07817.1997 9315902 PMC6793927

[glia24128-bib-0098] Pérez‐Otaño, I. , Larsen, R. S. , & Wesseling, J. F. (2016). Emerging roles of GluN3‐containing NMDA receptors in the CNS. Nature Reviews. Neuroscience, 17(10), 623–635. 10.1038/nrn.2016.92 27558536

[glia24128-bib-0099] Ramirez, S. , Liu, X. , Lin, P. A. , Suh, J. , Pignatelli, M. , Redondo, R. L. , Ryan, T. J. , & Tonegawa, S. (2013). Creating a false memory in the hippocampus. Science (New York, N.Y.), 341(6144), 387–391. 10.1126/science.1239073 23888038

[glia24128-bib-0100] Reilly, J. F. , Games, D. , Rydel, R. E. , Freedman, S. , Schenk, D. , Young, W. G. , Morrison, J. H. , & Bloom, F. E. (2003). Amyloid deposition in the hippocampus and entorhinal cortex: Quantitative analysis of a transgenic mouse model. Proceedings of the National Academy of Sciences of the United States of America, 100(8), 4837–4842. 10.1073/pnas.0330745100 12697936 PMC153642

[glia24128-bib-0101] Rolls, E. T. (1996). A theory of hippocampal function in memory. Hippocampus, 6(6), 601–620. 10.1002/(SICI)1098-1063(1996)6:6<601::AID-HIPO5>3.0.CO;2-J 9034849

[glia24128-bib-0102] Rosenmund, C. , & Stevens, C. F. (1996). Definition of the readily releasable pool of vesicles at hippocampal synapses. Neuron, 16(6), 1197–1207. 10.1016/s0896-6273(00)80146-4 8663996

[glia24128-bib-0103] Sahay, A. , Scobie, K. N. , Hill, A. S. , O'Carroll, C. M. , Kheirbek, M. A. , Burghardt, N. S. , Fenton, A. A. , Dranovsky, A. , & Hen, R. (2011). Increasing adult hippocampal neurogenesis is sufficient to improve pattern separation. Nature, 472(7344), 466–470. 10.1038/nature09817 21460835 PMC3084370

[glia24128-bib-0104] Santello, M. , Bezzi, P. , & Volterra, A. (2011). TNFα controls glutamatergic gliotransmission in the hippocampal dentate gyrus. Neuron, 69(5), 988–1001. 10.1016/j.neuron.2011.02.003 21382557

[glia24128-bib-0105] Santello, M. , Toni, N. , & Volterra, A. (2019). Astrocyte function from information processing to cognition and cognitive impairment. Nature Neuroscience, 22(2), 154–166. 10.1038/s41593-018-0325-8 30664773

[glia24128-bib-0106] Santello, M. , & Volterra, A. (2012). TNFα in synaptic function: Switching gears. Trends in Neurosciences, 35(10), 638–647. 10.1016/j.tins.2012.06.001 22749718

[glia24128-bib-0107] Sargolini, F. , Fyhn, M. , Hafting, T. , McNaughton, B. L. , Witter, M. P. , Moser, M. B. , & Moser, E. I. (2006). Conjunctive representation of position, direction, and velocity in entorhinal cortex. Science (New York, N.Y.), 312(5774), 758–762. 10.1126/science.1125572 16675704

[glia24128-bib-0108] Saunders, A. , Macosko, E. Z. , Wysoker, A. , Goldman, M. , Krienen, F. M. , De Rivera, H. , Bien, E. , Baum, M. , Bortolin, L. , Wang, S. , Goeva, A. , Nemesh, J. , Kamitaki, N. , Brumbaugh, S. , Kulp, D. , & McCarroll, S. A. (2018). Molecular diversity and specializations among the cells of the adult mouse brain. Cell, 174(4), 1015–1030.e16. 10.1016/j.cell.2018.07.028 30096299 PMC6447408

[glia24128-bib-0109] Savtchouk, I. , Di Castro, M. A. , Ali, R. , Stubbe, H. , Luján, R. , & Volterra, A. (2019). Circuit‐specific control of the medial entorhinal inputs to the dentate gyrus by atypical presynaptic NMDARs activated by astrocytes. Proceedings of the National Academy of Sciences of the United States of America, 116(27), 13602–13610. 10.1073/pnas.1816013116 31152131 PMC6612919

[glia24128-bib-0110] Savtchouk, I. , & Volterra, A. (2018). Gliotransmission: Beyond Black‐and‐white. The Journal of Neuroscience: The Official Journal of the Society for Neuroscience, 38(1), 14–25. 10.1523/JNEUROSCI.0017-17.2017 29298905 PMC6705815

[glia24128-bib-0111] Saylor, D. , Dickens, A. M. , Sacktor, N. , Haughey, N. , Slusher, B. , Pletnikov, M. , Mankowski, J. L. , Brown, A. , Volsky, D. J. , & McArthur, J. C. (2016). HIV‐associated neurocognitive disorder ‐ pathogenesis and prospects for treatment. Nature reviews. Neurology, 12(5), 309. 10.1038/nrneurol.2016.53 PMC5842923 27080521

[glia24128-bib-0112] Schwarz, M. K. , Scherbarth, A. , Sprengel, R. , Engelhardt, J. , Theer, P. , & Giese, G. (2015). Fluorescent‐protein stabilization and high‐resolution imaging of cleared, intact mouse brains. PLoS One, 10(5), e0124650. 10.1371/journal.pone.0124650 25993380 PMC4439039

[glia24128-bib-0113] Shigetomi, E. , Bushong, E. A. , Haustein, M. D. , Tong, X. , Jackson‐Weaver, O. , Kracun, S. , Xu, J. , Sofroniew, M. V. , Ellisman, M. H. , & Khakh, B. S. (2013). Imaging calcium microdomains within entire astrocyte territories and endfeet with GCaMPs expressed using adeno‐associated viruses. The Journal of General Physiology, 141(5), 633–647. 10.1085/jgp.201210949 23589582 PMC3639581

[glia24128-bib-0114] Shors, T. J. , Miesegaes, G. , Beylin, A. , Zhao, M. , Rydel, T. , & Gould, E. (2001). Neurogenesis in the adult is involved in the formation of trace memories. Nature, 410(6826), 372–376. 10.1038/35066584 11268214

[glia24128-bib-0115] Smith, T. D. , Adams, M. M. , Gallagher, M. , Morrison, J. H. , & Rapp, P. R. (2000). Circuit‐specific alterations in hippocampal synaptophysin immunoreactivity predict spatial learning impairment in aged rats. The Journal of Neuroscience: The Official Journal of the Society for Neuroscience, 20(17), 6587–6593. 10.1523/JNEUROSCI.20-17-06587.2000 10964964 PMC6772954

[glia24128-bib-0116] Solstad, T. , Boccara, C. N. , Kropff, E. , Moser, M. B. , & Moser, E. I. (2008). Representation of geometric borders in the entorhinal cortex. Science (New York, N.Y.), 322(5909), 1865–1868. 10.1126/science.1166466 19095945

[glia24128-bib-0117] Sorrells, S. F. , Paredes, M. F. , Cebrian‐Silla, A. , Sandoval, K. , Qi, D. , Kelley, K. W. , James, D. , Mayer, S. , Chang, J. , Auguste, K. I. , Chang, E. F. , Gutierrez, A. J. , Kriegstein, A. R. , Mathern, G. W. , Oldham, M. C. , Huang, E. J. , Garcia‐Verdugo, J. M. , Yang, Z. , & Alvarez‐Buylla, A. (2018). Human hippocampal neurogenesis drops sharply in children to undetectable levels in adults. Nature, 555(7696), 377–381. 10.1038/nature25975 29513649 PMC6179355

[glia24128-bib-0118] Sorrells, S. F. , Paredes, M. F. , Zhang, Z. , Kang, G. , Pastor‐Alonso, O. , Biagiotti, S. , Page, C. E. , Sandoval, K. , Knox, A. , Connolly, A. , Huang, E. J. , Garcia‐Verdugo, J. M. , Oldham, M. C. , Yang, Z. , & Alvarez‐Buylla, A. (2021). Positive controls in adults and children support that very few, if any, new neurons are born in the adult human hippocampus. The Journal of Neuroscience: The Official Journal of the Society for Neuroscience, 41(12), 2554–2565. 10.1523/JNEUROSCI.0676-20.2020 33762407 PMC8018729

[glia24128-bib-0119] Stark, R. , Grzelak, M. , & Hadfield, J. (2019). RNA sequencing: The teenage years. Nature Reviews. Genetics, 20(11), 631–656. 10.1038/s41576-019-0150-2 31341269

[glia24128-bib-0120] Stellwagen, D. , Beattie, E. C. , Seo, J. Y. , & Malenka, R. C. (2005). Differential regulation of AMPA receptor and GABA receptor trafficking by tumor necrosis factor‐alpha. The Journal of Neuroscience: The Official Journal of the Society for Neuroscience, 25(12), 3219–3228. 10.1523/JNEUROSCI.4486-04.2005 15788779 PMC6725093

[glia24128-bib-0121] Stellwagen, D. , & Malenka, R. C. (2006). Synaptic scaling mediated by glial TNF‐alpha. Nature, 440(7087), 1054–1059. 10.1038/nature04671 16547515

[glia24128-bib-0122] Stranahan, A. M. , & Mattson, M. P. (2010). Selective vulnerability of neurons in layer II of the entorhinal cortex during aging and Alzheimer's disease. Neural Plasticity, 2010, 108190. 10.1155/2010/108190 21331296 PMC3039218

[glia24128-bib-0123] Sun, M. Y. , Devaraju, P. , Xie, A. X. , Holman, I. , Samones, E. , Murphy, T. R. , & Fiacco, T. A. (2014). Astrocyte calcium microdomains are inhibited by bafilomycin A1 and cannot be replicated by low‐level Schaffer collateral stimulation in situ. Cell Calcium, 55(1), 1–16. 10.1016/j.ceca.2013.10.004 24262208

[glia24128-bib-0124] Sutton, M. A. , & Schuman, E. M. (2006). Dendritic protein synthesis, synaptic plasticity, and memory. Cell, 127(1), 49–58. 10.1016/j.cell.2006.09.014 17018276

[glia24128-bib-0125] Swardfager, W. , & Black, S. E. (2013). Dementia: A link between microbial infection and cognition? Nature Reviews. Neurology, 9(6), 301–302. 10.1038/nrneurol.2013.93 23712078

[glia24128-bib-0126] Tan, C. X. , & Eroglu, C. (2021). Cell adhesion molecules regulating astrocyte‐neuron interactions. Current opinion in neurobiology, 69, 170–177. 10.1016/j.conb.2021.03.015 33957433 PMC8387342

[glia24128-bib-0127] Turrigiano, G. G. (2008). The self‐tuning neuron: Synaptic scaling of excitatory synapses. Cell, 135(3), 422–435. 10.1016/j.cell.2008.10.008 18984155 PMC2834419

[glia24128-bib-0128] van Praag, H. , Kempermann, G. , & Gage, F. H. (1999). Running increases cell proliferation and neurogenesis in the adult mouse dentate gyrus. Nature Neuroscience, 2(3), 266–270. 10.1038/6368 10195220

[glia24128-bib-0129] Vyleta, N. P. , & Snyder, J. S. (2021). Prolonged development of long‐term potentiation at lateral entorhinal cortex synapses onto adult‐born neurons. PLoS One, 16(6), e0253642. 10.1371/journal.pone.0253642 34143843 PMC8213073

[glia24128-bib-0130] Wang, W. , Trieu, B. H. , Palmer, L. C. , Jia, Y. , Pham, D. T. , Jung, K. M. , Karsten, C. A. , Merrill, C. B. , Mackie, K. , Gall, C. M. , Piomelli, D. , & Lynch, G. (2016). A primary cortical input to hippocampus expresses a pathway‐specific and Endocannabinoid‐dependent form of long‐term potentiation. eNeuro, 3(4), 14. 10.1523/ENEURO.0160-16.2016 PMC4976302 27517090

[glia24128-bib-0131] Witter, M. P. (2007). The perforant path: Projections from the entorhinal cortex to the dentate gyrus. Progress in Brain Research, 163, 43–61. 10.1016/S0079-6123(07)63003-9 17765711

[glia24128-bib-0132] Witter, M. P. , & Moser, E. I. (2006). Spatial representation and the architecture of the entorhinal cortex. Trends in Neurosciences, 29(12), 671–678. 10.1016/j.tins.2006.10.003 17069897

[glia24128-bib-0133] Woods, N. I. , Vaaga, C. E. , Chatzi, C. , Adelson, J. D. , Collie, M. F. , Perederiy, J. V. , Tovar, K. R. , & Westbrook, G. L. (2018). Preferential Targeting of lateral Entorhinal inputs onto newly integrated granule cells. The Journal of Neuroscience: The Official Journal of the Society for Neuroscience, 38(26), 5843–5853. 10.1523/JNEUROSCI.1737-17.2018 29793975 PMC6021988

[glia24128-bib-0134] Yassa, M. A. , Muftuler, L. T. , & Stark, C. E. (2010). Ultrahigh‐resolution microstructural diffusion tensor imaging reveals perforant path degradation in aged humans in vivo. Proceedings of the National Academy of Sciences of the United States of America, 107(28), 12687–12691. 10.1073/pnas.1002113107 20616040 PMC2906542

[glia24128-bib-0135] Yoganarasimha, D. , Rao, G. , & Knierim, J. J. (2011). Lateral entorhinal neurons are not spatially selective in cue‐rich environments. Hippocampus, 21(12), 1363–1374. 10.1002/hipo.20839 20857485 PMC3010309

[glia24128-bib-0136] Zhang, Y. , Chen, K. , Sloan, S. A. , Bennett, M. L. , Scholze, A. R. , O'Keeffe, S. , Phatnani, H. P. , Guarnieri, P. , Caneda, C. , Ruderisch, N. , Deng, S. , Liddelow, S. A. , Zhang, C. , Daneman, R. , Maniatis, T. , Barres, B. A. , & Wu, J. Q. (2014). An RNA‐sequencing transcriptome and splicing database of glia, neurons, and vascular cells of the cerebral cortex. The Journal of Neuroscience: The Official Journal of the Society for Neuroscience, 34(36), 11929–11947. 10.1523/JNEUROSCI.1860-14.2014 25186741 PMC4152602

[glia24128-bib-0137] Zhou, Q. , Petersen, C. C. , & Nicoll, R. A. (2000). Effects of reduced vesicular filling on synaptic transmission in rat hippocampal neurones. The Journal of Physiology, 1(1), 195–206. 10.1111/j.1469-7793.2000.t01-1-00195.x PMC2269926 10811737

